# IL-6 prevents Th2 cell polarization by promoting SOCS3-dependent suppression of IL-2 signaling

**DOI:** 10.1038/s41423-023-01012-1

**Published:** 2023-04-12

**Authors:** Holly Bachus, Erin McLaughlin, Crystal Lewis, Amber M. Papillion, Etty N. Benveniste, Dave Durell Hill, Alexander F. Rosenberg, André Ballesteros-Tato, Beatriz León

**Affiliations:** 1 Department of Medicine, Division of Clinical Immunology and Rheumatology, University of Alabama at Birmingham, Birmingham, AL USA; 2 Department of Microbiology, University of Alabama at Birmingham, Birmingham, AL USA; 3 Department of Cell, Developmental and Integrative Biology, University of Alabama at Birmingham, Birmingham, AL USA; 4 Informatics Institute, University of Alabama at Birmingham, Birmingham, AL USA; 5 Present Address: Alexion Pharmaceuticals, Inc., New Haven, CT USA

**Keywords:** Th2 cell polarization, IL-6, IL-2, SOCS3, JAK1 inhibitor, Allergy, Lymphocyte differentiation

## Abstract

Defective interleukin-6 (IL-6) signaling has been associated with Th2 bias and elevated IgE levels. However, the underlying mechanism by which IL-6 prevents the development of Th2-driven diseases remains unknown. Using a model of house dust mite (HDM)-induced Th2 cell differentiation and allergic airway inflammation, we showed that IL-6 signaling in allergen-specific T cells was required to prevent Th2 cell differentiation and the subsequent IgE response and allergic inflammation. Th2 cell lineage commitment required strong sustained IL-2 signaling. We found that IL-6 turned off IL-2 signaling during early T-cell activation and thus inhibited Th2 priming. Mechanistically, IL-6-driven inhibition of IL-2 signaling in responding T cells was mediated by upregulation of Suppressor Of Cytokine Signaling 3 (SOCS3). This mechanism could be mimicked by pharmacological Janus Kinase-1 (JAK1) inhibition. Collectively, our results identify an unrecognized mechanism that prevents the development of unwanted Th2 cell responses and associated diseases and outline potential preventive interventions.

## Introduction

Interleukin-6 (IL-6) is a pleiotropic cytokine that plays critical roles in regulating inflammation, hematopoiesis, metabolism, and oncogenesis [1]. Classic IL-6 signaling is initiated by the binding of IL-6 to the membrane-bound IL-6-specific receptor α chain (IL-6R). The IL-6/IL-6R complex triggers its association with the signal-transducing subunit, glycoprotein 130 (GP130), leading to the phosphorylation of Janus Kinases (JAKs) and Signal Transducers and Activator of Transcription (STATs), which culminates in the nuclear import of phosphorylated STAT dimers, predominantly STAT3 dimers, that activate transcription [2]. IL-6 signaling is negatively regulated by Suppressors Of Cytokine Signaling (SOCS) proteins, which are generally negative-feedback inhibitors of signaling induced by cytokines that act via the JAK/STAT pathway [3].

Excess IL-6 is central to the pathogenesis of multiple inflammatory conditions, such as rheumatoid diseases, cytokine storm, and cytokine release syndrome, and targeting the IL-6 pathway has led to innovative therapeutic approaches for some of these inflammatory diseases [4]. On the other hand, loss-of-function mutations that affect IL-6 signaling, including mutations in IL6R [5], GP130 [6, 7], and STAT3 [8–11], lead to increased T helper 2 (Th2) bias and manifestation of hallmarks of Th2-mediated immune responses, such as high serum allergen-specific and total IgE concentrations and eosinophilia [12]. Consequently, patients with these mutations often present with atopy and cutaneous, airway, and systemic manifestations of allergy [13]. Although studies of patients have established a role for IL-6 in controlling Th2 bias, the specific contribution of IL-6 and the underlying mechanism remain undefined.

Using a house dust mite (HDM)-induced model of Th2-driven allergic airway inflammation, we show that IL-6 signaling in allergen-specific T cells is required to suppress Th2 cell lineage commitment. Although T-bet has been shown to suppress the Th2 cell-associated program [14, 15], we found that suppression of allergen-specific Th2 cell responses by IL-6 was independent of T-bet. Instead, we found that Th2 cell lineage commitment required strong and prolonged IL-2 signaling but that IL-6 shut down IL-2 signaling during early T-cell activation and thus inhibited Th2 cell priming. Mechanistically, IL-6 upregulated SOCS3, thus preventing prolonged IL-2 signaling through STAT5 and Th2 cell programming. Furthermore, we found that inhibition of JAK1 with a selective inhibitor could likewise prevent sustained IL-2 signaling and Th2 cell differentiation.

Collectively, our data describe an unrecognized mechanism that prevents the development of harmful Th2 cell responses and allergic disorders. In addition, our data define the specific role of IL-6 signaling in this process. Understanding how IL-6 contributes to allergic sensitization may offer new strategies to prevent atopic disease in patients with deficient IL-6 signaling.

## Results

### IL-6 is required to suppress allergen-specific Th2 cell responses

**Fig. 1:**
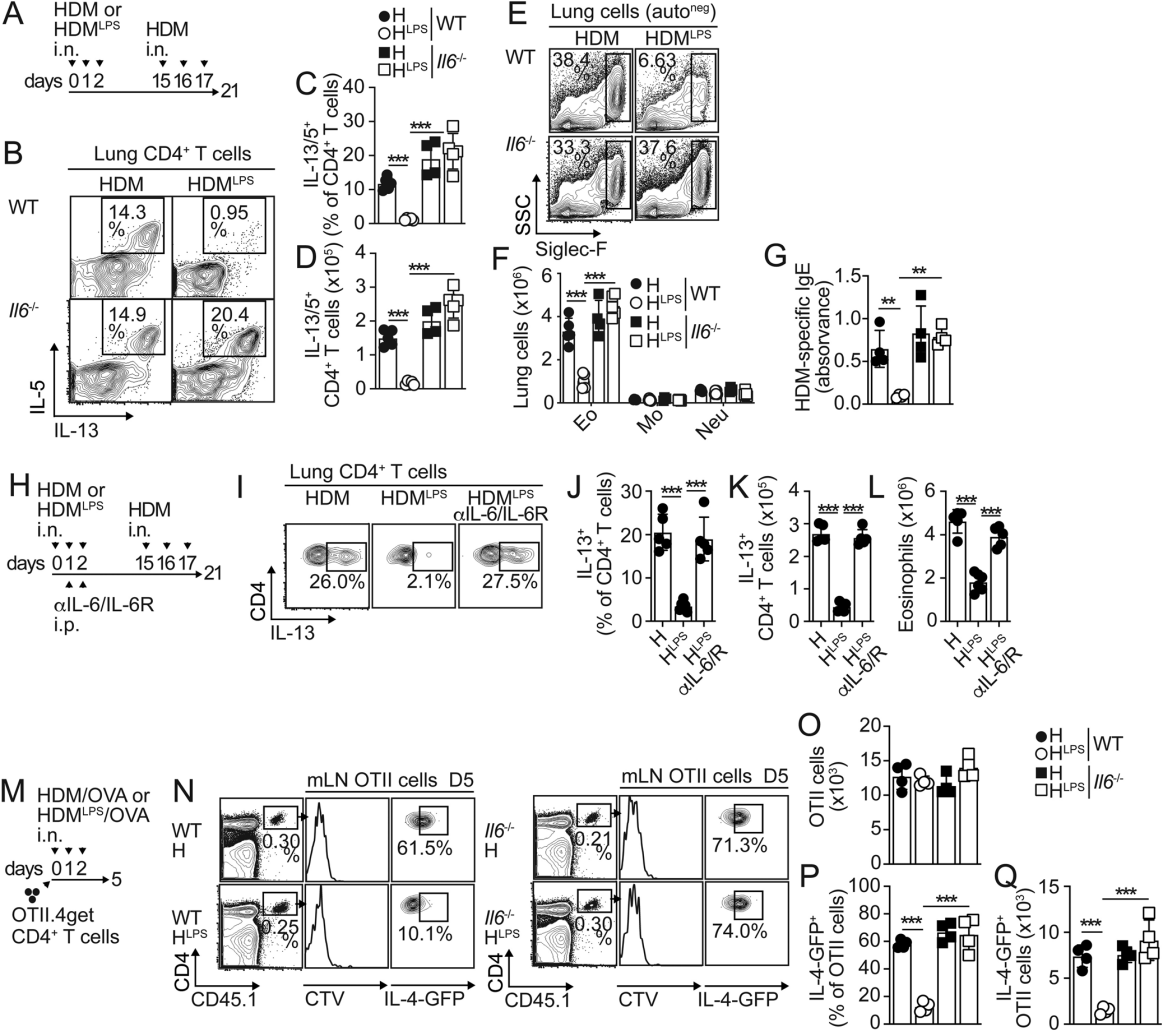
IL-6 signaling during allergen sensitization suppresses allergen-specific Th2 cell-mediated immunity. **A–G** B6 (WT) and *Il6*^*-/-*^ mice were i.n. treated with 100 µg low-endotoxin HDM (HDM; endotoxin content <30 EU/mg) or high-endotoxin HDM (HDM^LPS^; endotoxin content: 1 µg/mg, ~250 EU/mg) for 3 days. On Day 15, the mice were i.n. challenged with 100 µg HDM daily for 3 days and then analyzed on Day 21 (**A**). Frequencies (**B, C**) and numbers (**D**) of IL-13^+^IL-5^+^ CD4^+^ T cells in the lungs. Frequencies and numbers of eosinophils, neutrophils and monocytes in the lungs (**E, F**). HDM-specific IgE levels in the serum (**G**). **H–L** B6 mice were i.n. sensitized with HDM or HDM^LPS^. Some mice also received 250 µg anti-IL-6 and anti-IL-6R (i.p.). On Day 15, the mice were i.n. challenged with HDM and then analyzed on Day 21 (**H**). Frequencies (**I, J**) and numbers (**K**) of IL-13^+^ CD4^+^ T cells in the lungs. Numbers of lung eosinophils (**L**). **M–Q** Mice were transferred with OTII.4get cells, i.n. treated with HDM or HDM^LPS^ + 5 µg OVA for 3 days and analyzed on Day 5. Frequencies and numbers of total (**N–O**) and IL-4-GFP^+^ (**N, P, Q**) OTII cells in the mLN. Data are representative of at least three independent experiments (mean±S.D., *n* = 4-5, two-way and one-way ANOVA). See Fig. S1

To test whether IL-6 influences the Th2 cell response to HDM, we intranasally (i.n.) sensitized and challenged WT and *Il6*^−/−^ mice with HDM (Fig. 1A) and quantified IL-13/IL-5^+^ Th2 cells in the lungs. We found robust lung accumulation of Th2 cells, but there were no differences between WT and *Il6*^*−/−*^ mice (Fig. 1B, D). In nature, dust allergens are often contaminated with lipopolysaccharide/endotoxin (LPS) at variable levels from ~10 to 1000 EU/mg [16, 17], with higher levels helping to prevent Th2 cell priming and the development of allergic inflammation [15, 18–21]. Our HDM extract contained low endotoxin levels (<30 endotoxin units (EU)/mg). By adding LPS (1 µg LPS per 1 mg HDM), we generated high-endotoxin HDM (HDM^LPS^; endotoxin content ~250 EU/mg) and used it to sensitize mice (Fig. 1A). HDM^LPS^ sensitization elevated the IL-6 levels in bronchoalveolar lavage fluid (BALF) compared with HDM sensitization (Fig. S1A). As expected, sensitization with HDM^LPS^ prevented the accumulation of Th2 cells in the lungs of challenged WT mice (Fig. 1B, D) [18]. Importantly, however, HDM^LPS^ exposure failed to prevent the increase in Th2 cells in *Il6*^*−/−*^ mice. As a result, Th2 cells largely accumulated in the lungs of HDM^LPS^-sensitized *Il6*^*−/−*^ compared to those of WT mice (Fig. 1B–D). These data show that IL-6 is required to prevent the accumulation of allergen-induced Th2 cells. In agreement, while sensitization with HDM^LPS^ prevented Th2-driven eosinophilic airway inflammation (Fig. 1E, F) and serum HDM-specific IgE increase (Fig. 1G) in challenged WT mice, it failed to do so in *Il6*^*−/−*^ mice. No differences were observed in lung monocytes or neutrophils (Fig. 1F and S1B), although *Il6*^*−/−*^ mice showed significantly less accumulation of Th17 cells in the lungs (Fig. S1C–E). Our analysis also found no differences in lung regulatory T cells (Tregs) (Fig. S1F-H). Similar results were obtained when IL-6 signaling was blocked using neutralizing antibodies against IL-6 and IL-6R during sensitization (Fig. 1H–L and S1I–L).

The development of allergen-specific effector Th2 cells in the lungs requires initial priming of T cells in the lung-draining mediastinal LN (mLN) [22]. To analyze differences in T-cell priming between WT and *Il6*^*−/−*^ mice, we transferred IL-4-GFP reporter (4get) OTII TCR-transgenic CD4^+^ T cells, followed by sensitization with HDM or HDM^LPS^ in the presence of OVA (Fig. 1M). No differences in the expansion of donor OTII cells in the mLN (Fig. 1N–O) or in the accumulation of endogenous Tregs (Fig. S1M–O) were observed between the groups. As expected, HDM^LPS^ sensitization prevented the accumulation of IL-4-GFP^+^4get.OTII cells in WT recipients. In contrast, HDM^LPS^ did not prevent the increase in GFP^+^CD4^+^ T- cell accumulation in *Il6*^*−/−*^ mice **(**Figs. 1N and 1P, Q**)**. Consequently, GFP^+^4get.OTII cells failed to accumulate in the lungs of HDM^LPS^-sensitized WT mice after challenge but largely accumulated in the lungs of HDM^LPS^-sensitized *Il6*^*−/−*^ mice (Fig. S1Q–T**)**. Taken together, these data indicate that IL-6, which is produced in response to endotoxin-contaminated HDM, is required to prevent the development of specific Th2 cell responses and subsequent allergic inflammation. Notably, the B6 background was used in the present study, and mice on this background showed a greater response to LPS than BALB/c mice. BALB/c mice required higher doses of LPS to prevent allergen-induced Th2 cell accumulation (Fig. S1U**)**, but blockade of IL-6 signaling counteracted this effect as in B6 mice (Fig. S1V**)**.

### IL-6 signaling in allergen-specific T cells is required to suppress Th2 lineage commitment

**Fig. 2:**
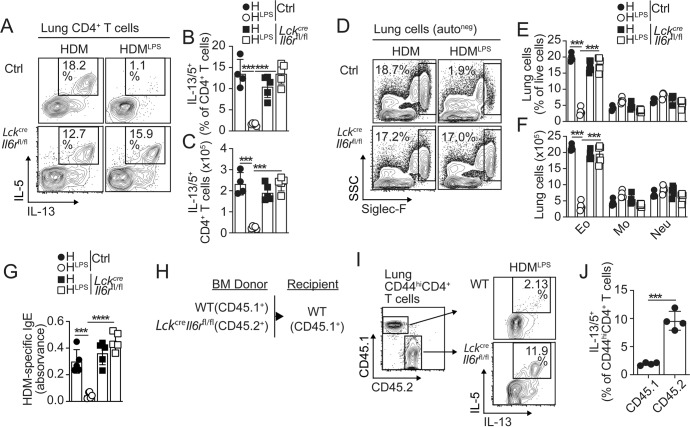
IL-6 signaling in allergen-specific T cells suppresses Th2 cell polarization. (**A-G**) *Lck*^*cre*^*-Il6r*^*fl/fl*^ and control mice were i.n. sensitized with HDM or HDM^LPS^ and challenged with HDM. Frequencies (**A, B**) and numbers (**C**) of IL-13^+^IL-5^+^ CD4^+^ T cells in the lungs. Frequencies and numbers of eosinophils, neutrophils and monocytes in the lungs (**D–F**). HDM-specific IgE levels in the serum (**G**). **H–J** Irradiated B6 (CD45.1^+^) mice were reconstituted with a 1:1 BM mix from B6 (CD45.1^+^) and *Lck*^*cre*^*-Il6r*^*fl/fl*^ (CD45.2^+^) donors (**G**). Chimeras were i.n. sensitized with HDM^LPS^ and challenged with HDM. Frequencies of IL-13^+^IL-5^+^ WT and *Il6r*^−/−^ CD44^hi^CD4^+^ T cells in the lungs (**I, J**). Data are representative of at least three independent experiments (mean±S.D., *n* = 4-5, two-way ANOVA and unpaired Student’s *t* test). See Fig. S2

We next determined whether IL-6 signaling in T cells is required for the suppression of Th2 cell responses. Thus, we sensitized and challenged *Lck*^*cre*^*-Il6r*^*fl/fl*^ and control mice and analyzed cytokine production by T cells in the lungs. Th2 cells failed to accumulate in the lungs of control mice that were sensitized with HDM^LPS^. Importantly, however, HDM^LPS^ exposure was unable to prevent the expansion of Th2 cells in *Lck*^*cre*^*-Il6r*^*fl/fl*^ mice (Fig. 2A–C). Consequently, eosinophilia (Fig. 2D–F) and serum HDM-specific IgE levels (Fig. 2G) were suppressed in HDM^LPS^-treated control mice but not in *Lck*^*cre*^*-Il6r*^*fl/fl*^ mice. Our model of sensitization and challenge did not induce significant numbers of IFNγ^+^CD4^+^ T cells in the lungs, although it induced IL-17 production by T cells (Fig. 2SA–E). We found that CD4^+^, T cells from *Lck*^*cre*^*-Il6r*^*fl/fl*^ mice produced significantly less IL-17 (Fig. S2A–C), but no differences in lung neutrophilic or monocytic inflammation were observed between *Lck*^*cre*^*-Il6r*^*fl/fl*^ and control mice (Fig. 2E, F and S2F). IL-6 has been identified as a master regulator of IL-21 in T cells [23, 24]. Thus, we studied whether IL-6-driven inhibition of Th2 cell responses was mediated by secondary IL-21 production and signaling. WT and *Il21r*^*−/−*^ mice were sensitized and challenged, and the T-cell and inflammatory responses in the lungs were analyzed. HDM^LPS^ sensitization similarly prevented the accumulation of IL-13/IL-5^+^, Th2 cells (Fig. S2G–I) and eosinophils (Fig. S2J–L) in WT and *Il21r*^*−/−*^ mice. Furthermore, no differences in IL-17 production by T cells were found (Fig. S2M–O). Thus, IL-21 signaling is not required to prevent the development of Th2 cell responses to HDM, while IL-6 signaling is.

We finally assessed whether the requirement for IL-6 signaling is intrinsically necessary in HDM-responsive CD4^+^ T cells. To do this, we sensitized WT:*Lck*^*cre*^*-Il6r*^*fl/fl*^ mixed bone marrow (BM) chimeras (Fig. 2H) with HDM^LPS^ and challenged them with HDM. We determined the frequency of Th2 cells within the WT and *Il6r*^−/−^ CD44^hi^CD4^+^ T-cell compartments (Fig. 2I) and found that Th2 cells largely accumulated in the *Il6r*^*−/−*^ compartment compared to the WT compartment (Fig. 2J). These results indicated that IL-6 signaling in responding T cells was intrinsically required to suppress the Th2 cell differentiation program.

### Suppression of allergen-specific Th2 cell responses by IL-6 is T-bet independent

**Fig. 3:**
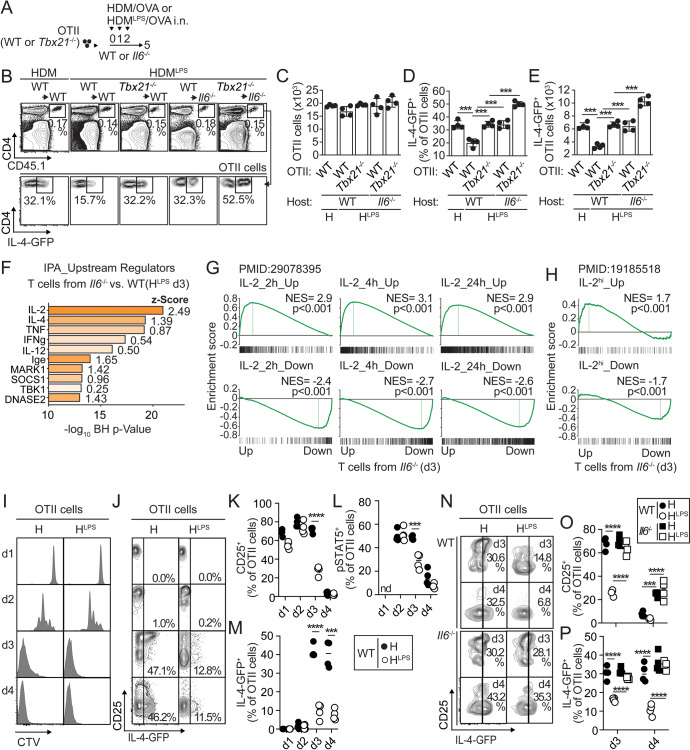
IL-6 signaling in responder T cells prevents prolonged IL-2 responsiveness. **A–E** WT and *Il6*^*-/-*^ mice were transferred with WT or *Tbx21*^−/−^ OTII.4get cells, i.n. treated with HDM or HDM^LPS^ + OVA for 3 days and analyzed on Day 5 (**A**). Frequencies and numbers of total (**B, C**) and IL-4-GFP^+^ (**B, D, E**) OTII cells in the mLN. (**F–H**) WT and *Il6*^*-/-*^ mice were transferred with OTII cells and i.n. sensitized with HDM^LPS^ + OVA. On Day 3, OTII cells were sorted from the mLN, and RNA-seq was performed (three replicates). A total of 317 differentially expressed genes, with 103 up- and 214 downregulated genes in OTII cells harvested from *Il6*^*-/-*^ mice, were identified (FDR < 0.05, ≥2 FC. See Table S1). Activated upstream regulators (positive Z score) in OTII cells from *Il6*^*-/-*^ versus WT mice based on IPA (**F**). GSEA plots showing the enrichment of genes regulated by IL-2 (**G**) or by strong IL-2 signaling (**H**) in OTII cells from *Il6*^*-/-*^ versus WT mice. (**I–P**) WT (**I–P**) and *Il6*^*-/-*^ (**N–P**) mice were transferred with CTV-labeled OTII.4get cells and i.n. sensitized with HDM or HDM^LPS^ + OVA. CTV profiles (**I**) and frequencies of CD25^+^ (**K, O**) and IL-4-GFP^+^ cells (**J, M, N, P**) in donor OTII cells from the mLNs on different days. Cells from the mLNs were stimulated with 1 µg/ml rIL-2 for 15 min, and STAT5 phosphorylation in OTII cells was determined (**L**). Data are representative of two independent experiments (mean±S.D., *n* = 3-4, one-way and two-way ANOVA and unpaired Student’s *t* test). See Fig. S3 and S4

T-bet suppresses the Th2 cell differentiation program to HDM [15]. Thus, we tested whether IL-6 signaling suppresses Th2 cell differentiation via T-bet. We transferred WT (CD45.1^+^) or Tbx21^−/−^ (CD45.1^+^) 4get.OT-II cells into CD45.2^+^ WT and *Il6*^*−/−*^ recipients, sensitized the recipient mice and analyzed the expanded WT and *Tbx21*^*−/−*^ donor cells (Fig. 3A). OT-II cells accumulated similarly in all the groups (Fig. 3B, C). A deficiency in T-bet or IL-6 signaling counteracted the effect of HDM^LPS^ in preventing IL-4-GFP expression in OTII cells (Figs. 3B, D) and the consequent accumulation of GFP^+^4get.OTII cells (Fig. 3E). We found, however, that combining both deficiencies had an additive effect and promoted a greater accumulation of GFP^+^4get.OTII cells than when these deficiencies occurred separately (Fig. 3E). These data suggest that T-bet expression and IL-6 signaling play independent roles in suppressing the Th2 cell differentiation program in response to HDM.

We found, however, that IL-6 (Fig. S3A–G) and IL-6 signaling (Fig. S3H–M) were dispensable for inhibiting lung Th2 cell accumulation and allergic eosinophilic inflammation in response to HDM contaminated with 400-fold higher levels of LPS (i.e., 0.4 mg LPS per 1 mg HDM; HDM^LPS400^). Additionally, IL-6 was not required to inhibit the lung accumulation of HDM-induced endogenous (Fig. S3O–Q) or OTII donor (Fig. S3R–V) Th2 cells when T cells were primed in an IL-12-rich environment. Both HDM^LPS400^ and exogenous IL-12 induced high levels of T-bet expression in responding T cells (not shown). Thus, IL-6 signaling is particularly necessary to suppress the Th2 cell differentiation program under reduced T-bet/Th1-polarizing conditions.

### IL-6 suppresses IL-2 signaling early after T-cell activation

To understand the role of IL-6 in suppressing Th2 cell commitment, we performed RNA-seq to compare the transcriptomes of donor OT-II cells primed in WT and *Il6*^*−/−*^ recipients after sensitization with HDM^LPS^ + OVA. A total of 317 genes, defined by an FDR < 0.05 and at least a 2-fold change, were differentially expressed between OTII cells primed in *Il6*^*−/−*^ and WT recipients on Day 3 (Table S1). Ingenuity Pathway Analysis (IPA) predicted that IL-2 was the regulator with the highest significant enrichment and the largest activation z score (-log (*p-value*)=22, Z Score=2.5) (Fig. 3F and Table S2). Furthermore, a previously identified kinetic cluster of genes regulated by IL-2 [25] as well as highly inducible IL-2 genes [26] in CD4^+^ T cells were highly enriched in OTII cells primed in *Il6*^*−/−*^ mice (Fig. 3G, H). These data suggest dominant regulation by IL-2 in the absence of IL-6 signaling. The highest normalized enrichment scores (NES) were found for T cells from Day 3 compared with those from Day 5 (Fig. 3G, H and S4A, B), suggesting the most prominent IL-2-dependent regulation occurred early after T-cell activation. In agreement, we found that the gene encoding the IL-2 receptor α chain (*Il2ra*), which is the top-ranked gene regulated by IL-2 [25], was highly expressed in OTII cells from HDM^LPS^-sensitized *Il6*^*−/−*^ recipients, particularly at Day 3 but not Day 5 (Fig. S4C). No differences were found in the expression of the IL-2 receptor β chain (*Il2rb*) (Fig. S4D). These data suggested that IL-6 inhibited IL-2 signaling early after T-cell activation and thus prevented IL-2-dependent gene regulation.

To test the prediction that IL-6-dependent inhibition of IL-2 signaling in T cells is required to suppress the Th2 cell differentiation program in response to HDM, we first analyzed the kinetics of OTII cell activation in WT recipients following sensitization with HDM or HDM^LPS^ in the presence of OVA. On Day 1 after treatment, OTII cells had not yet proliferated (Fig. 3I) but exhibited strong upregulation of IL-2Rα/CD25 expression (Fig. 3J, K), suggesting robust IL-2 signaling. However, no differences were found between sensitization with HDM and HDM^LPS^. On Day 2, OTII cells from HDM- and HDM^LPS^-sensitized mice began proliferating, as shown by CellTrace Violet (CTV) dilution (Fig. 3I), and still maintained strong responsiveness to IL-2, as indicated by elevated expression of IL-2-driven pSTAT5 (Fig. 3L) and CD25 (Fig. 3J-K). On Day 3, OTII cells from HDM-sensitized mice began to increase IL-4 expression (Figs. 3J–M). OTII cells still retained strong responsiveness to IL-2 (Fig. 3J–L), and indeed, OTII cells expressing the highest levels of CD25 upregulated IL-4 expression (Fig. 3J). In contrast, OTII cells sensitized in the presence of HDM^LPS^ did not prominently upregulate IL-4 (Figs. 3J–M), coinciding with the early downregulation of CD25 expression beginning on Day 3 (Fig. 3J, K) and poor responsiveness to IL-2 (Fig. 3L). These results showed that HDM^LPS^ sensitization limited IL-2/STAT5 responsiveness shortly after T-cell activation and before Th2 cell commitment. To test whether limited IL-2/STAT5 responsiveness in T cells after HDM^LPS^ sensitization is dependent on the presence of IL-6, we performed similar experiments with *Il6*^*−/−*^ recipients. OTII cells proliferated similarly in WT and *Il6*^*−/−*^ recipients (Fig. S4E). However, we found that the early downregulation of CD25 expression (Fig. 3N, O) and premature attenuation of IL-2-driven pSTAT5 expression (Fig. S4F) observed in OTII cells from WT mice on Day 3 post-HDM^LPS^ sensitization did not occur in *Il6*^*−/−*^ recipients. Consistent with these results, OTII cells in HDM^LPS^-sensitized *Il6*^*−/−*^ mice did not suppress IL-4 expression (Fig. 3P). Our analyses did not find differences in the IL-2-driven pSTAT5 response in endogenous Tregs (Fig. S4G). Collectively, these results show that IL-6 limits IL-2 signaling in antigen-specific T cells and suggest that restricting IL-2 signaling is a mechanism to suppress the Th2 cell differentiation program in response to HDM.

### IL-6 prevents IL-2 responsiveness required for the polarization of naïve T cells to the Th2 cell phenotype

**Fig. 4:**
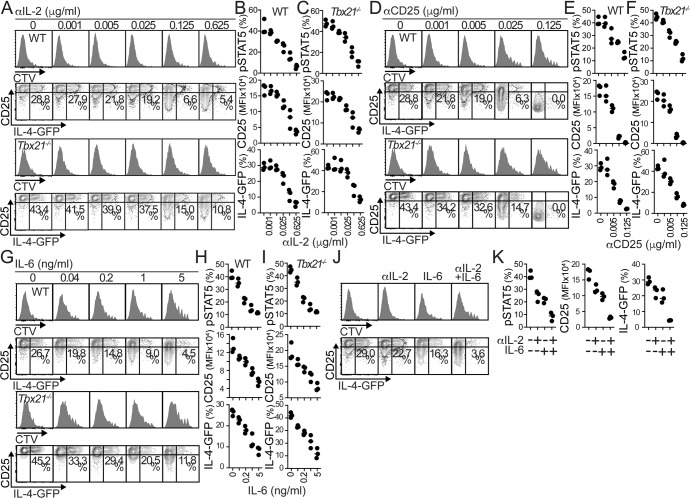
Th2 cell polarization requires strong IL-2 signaling, which is prevented by IL-6. **A–I** CTV-labeled WT.4get (CD45.1^+^) and *Tbx21*^−/−^.4get (CD45.2^+^) CD4^+^ T cells from naïve mice were mixed 1:1 and stimulated in vitro with plate-bound anti-CD3 and soluble anti-CD28 for 48 hours. Anti-IL-2 Abs (JES6-1A12 and S4B6), anti-CD25 (PC-61.5.3), or rIL-6 was added at the indicated concentrations for an additional 72 hours. CTV profiles and frequencies of pSTAT5^+^, CD25^+^, and IL-4-GFP^+^ cells in WT (**A, B, D–E, G, H**) and *Tbx21*^−/−^ (**A, C, D, F, G, I**) CD4^+^ T cells. **J, K** CTV-labeled WT.4get CD4^+^ T cells from naïve mice were activated in vitro with plate-bound anti-CD3 and soluble anti-CD28 for 48 hours. Anti-IL-2 Abs (0.025 □g/ml), rIL-6 (0.2 ng/ml), or both were added for an additional 72 hours. CTV profiles (**J**) and frequencies of pSTAT5^+^, CD25^+^, and IL-4-GFP^+^ cells **J, K**. Data are representative of four independent experiments. All values were obtained in triplicate, and the data are shown as the mean±S.D

IL-2 plays a critical role in the polarization of Th2 cells [27–29]. Consequently, we found that in vitro neutralization of IL-2 reduced the frequency of IL-4-producing cells in CD3/CD28-stimulated 4get.CD4^+^ T cells without altering cell division (Fig. 4A). In control conditions, IL-4-GFP was produced by CD25^hi^ CD4^+^ T cells, but anti-IL-2 reduced the expression of pSTAT5 and CD25 concurrently with the reduction in IL-4-GFP production (Fig. 4A, B). IL-2 neutralization similarly inhibited IL-4-GFP expression by WT (Fig. 4A, B) and *Tbx21*^*−/−*^ (Figs. 4A and 4C) CD4^+^ T cells. Comparable results were observed when the strength of IL-2 signaling in T cells was inhibited by blocking CD25 (Fig. 4D–F). Thus, strong IL-2 signaling in activated CD4^+^ T cells is required to generate IL-4 producers.

To test whether the presence of IL-6 affects IL-2 responsiveness and IL-4 expression, CD3/CD28-stimulated 4.get.CD4^+^ T cells were cultured in the presence of increasing concentrations of rIL-6. CD4^+^ T lymphocytes proliferated at a similar rate except at high concentrations of IL-6, which partially suppressed proliferation. Furthermore, we found that the addition of IL-6 reduced the expression of pSTAT5, CD25, and IL-4-GFP in WT (Fig. 4G, H) and *Tbx21*^*−/−*^ (Figs. 4G, I) CD4^+^ T cells. These results indicated that IL-6 signaling prevented IL-2 responsiveness and thus early IL-4 production by activated and proliferating CD4^+^ T cells. These findings also indicated that these IL-6-dependent functions were independent of T-bet expression. To confirm these conclusions, we tested whether the presence of IL-6 affects the threshold of anti-IL-2 required to prevent IL-4 production. The addition of low concentrations of anti-IL-2 or rIL-6 to stimulate 4.get.CD4^+^ T cells moderately decreased the expression of pSTAT5, CD25, and IL-4-GFP (Fig. 4J, K). However, combining the treatments greatly reduced IL-2 responsiveness in T cells and largely suppressed IL-4-GFP expression (Fig. 4J, K). Thus, in the presence of IL-6, low concentrations of anti-IL-2 can successfully block the response to IL-2 and Th2 cell priming, reinforcing the conclusion that IL-6 controls IL-2 signaling in T cells and, as such, suppresses the priming needed for Th2 cell differentiation.

### In vivo blockade of IL-2 signaling suppresses allergen-specific Th2 cell responses occurring in the absence of IL-6 signaling

**Fig. 5:**
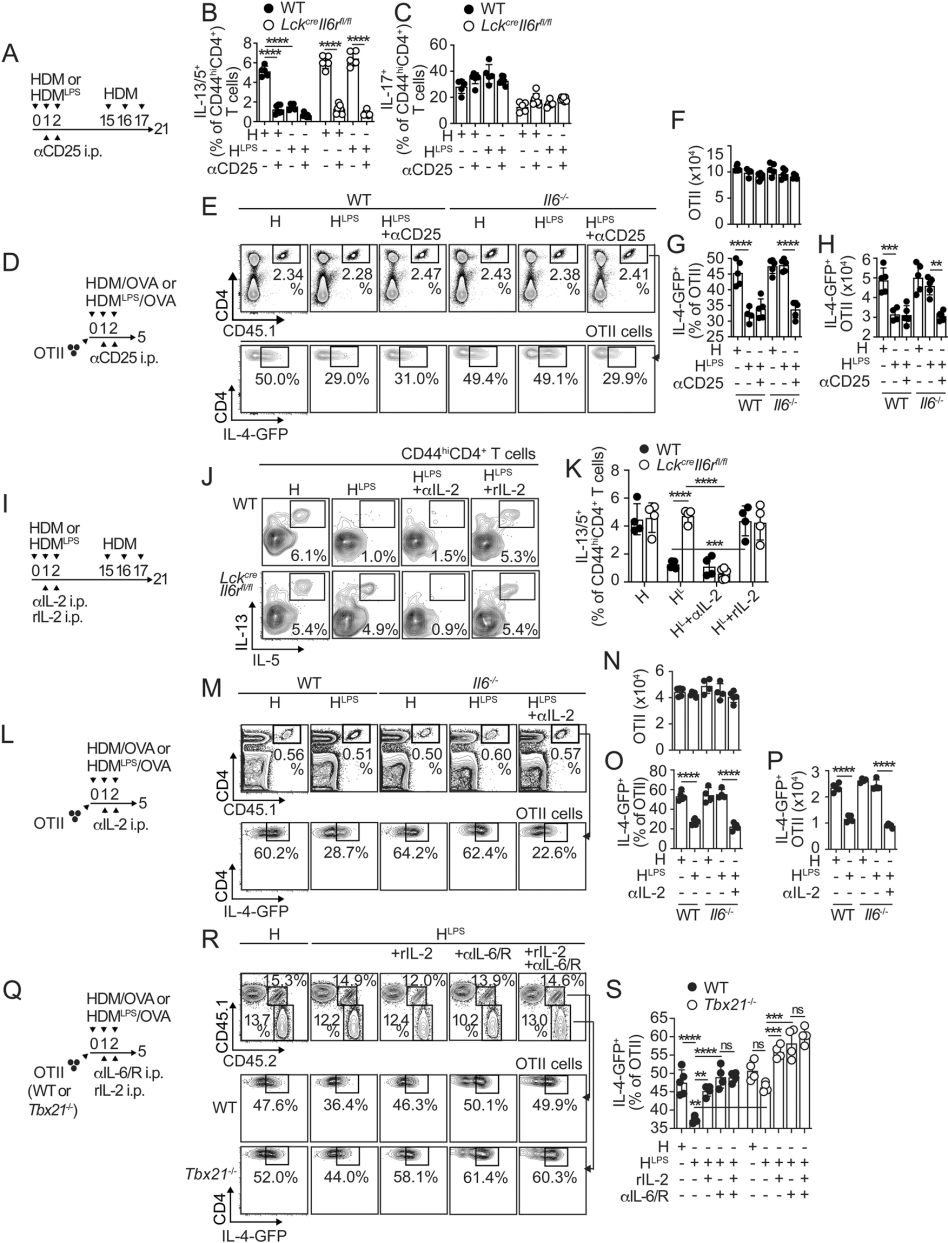
IL-6 and IL-2 signaling in allergen-specific T cells oppositely regulate polarization toward a Th2 profile. **A–C** WT:*Lck*^*cre*^*-Il6r*^*fl/fl*^ chimeras were i.n. sensitized with HDM or HDM^LPS^, i.p. treated with anti-CD25 or PBS, and i.n. challenged with HDM (**A**). Frequencies of IL-13^+^IL-5^+^ (**B**) and IL-17^+^ (**C**) cells in the WT and *Lck*^*cre*^*-Il6r*^*fl/fl*^ CD44^hi^CD4^+^ T-cell compartments in the lungs. **D–H** WT and *Il6*^*-/-*^ mice were transferred with OTII.4get cells, i.n. treated with HDM or HDM^LPS^ + OVA, and i.p. treated with anti-CD25 or PBS (**D**). Frequencies and numbers of total (**E, F**) and IL-4-GFP^+^ (**E, G, H**) OTII cells in the mLN. (**I–K**) WT:*Lck*^*cre*^*-Il6r*^*fl/fl*^ chimeras were i.n. sensitized with HDM or HDM^LPS^; i.p. treated with anti-IL-2 Abs (JES6-1A12 and S4B6; 250 µg each), rIL-2 (60,000 U) or PBS; and i.n. challenged with HDM (**I**). Frequencies of IL-13^+^IL-5^+^ cells in the WT and *Lck*^*cre*^*-Il6r*^*fl/fl*^ CD44^hi^CD4^+^ T-cell compartments in the lungs (**J, K**). **L–P** WT and *Il6*^*-/-*^ mice were transferred with OTII.4get cells, i.n. treated with HDM or HDM^LPS^ + OVA, and i.p. treated with anti-IL-2 Abs or PBS (**L**). Frequencies and numbers of total (**M, N**) and IL-4-GFP^+^ (**M, O, P**) OTII cells in the mLN. **Q–S** WT (CD45.1^+^) mice were cotransferred with WT (CD45.1^+^CD45.2^+^) and *Tbx21*^−/−^ (CD45.2^+^) OTII.4get cells, i.n. treated with HDM or HDM^LPS^ + OVA, and i.p. treated with anti-IL-6 and anti-IL-6R Abs, rIL-2 or PBS. Frequencies of total (**R**) and IL-4-GFP^+^ (**R, S**) WT and *Tbx21*^−/−^ OTII cells in the mLN. Data are representative of two independent experiments (mean±S.D., *n* = 4-6, one-way ANOVA). See Fig. S5

We next studied whether in vivo inhibition of IL-2 signaling can suppress Th2 cell responses to HDM in low-IL-6 environments. Thus, we blocked the binding of IL-2 to its receptor by administering an anti-CD25 mAb to WT:*Lck*^*cre*^*-Il6r*^*fl/fl*^ (*Il6r*^*-/-*^) mixed BM chimeras following initial sensitization (Fig. 5A). We found similar frequencies and numbers of WT and *Il6r*^*-/-*^ CD44^hi^CD4^+^ T cells in the lungs under all the treatments (Fig. S5A, B). As expected, HDM sensitization and challenge induced robust lung accumulation of Th2 cells from both WT and *Il6r*^*-/-*^ donors. However, anti-CD25 treatment prevented this Th2 cell accumulation (Fig. 5B), indicating that effector Th2 cell responses to HDM depended on strong IL-2 signaling. Furthermore, anti-CD25 treatment effectively prevented Th2 cell development in *Il6r*^*-/-*^ T cells from HDM^LPS^-sensitized mice (Fig. 5B). Treatment with anti-CD25 did not affect Tregs numbers (Fig. S5C). It also did not affect IL-17 production by activated CD4^+^ T cells, although, as expected, *Il6r*^*-/-*^ T cells produced less IL-17 (Fig. 5C). These data show that blocking IL-2 signaling can prevent Th2 cell development in settings with low IL-6 stimulation.

We confirmed that blocking IL-2 signaling suppressed the priming of antigen-specific Th2 cells by analyzing donor 4get.OTII cells in the mLN of WT and *Il6*^-/-^ recipients after sensitization (Fig. 5D). Consistent with our previous results, an anti-CD25 mAb administered 24 h after sensitization did not affect the expansion of donor OTII cells (Fig. 5E, F) but suppressed their IL-4-GFP^+^ expression and thus the accumulation of IL-4-GFP^+^4get.OTII cells in HDM^LPS^-sensitized *Il6*^-/-^ recipients (Fig. 5E, G, H).

In addition, we examined the response of *Il2ra*^*-/-*^ CD4^+^ T cells, which cannot transduce normal IL-2 signals. Thus, WT:*Il2ra*^*-/-*^ mixed BM chimeras were sensitized, treated with anti-IL-6/IL-6R or a control mAb, and analyzed after challenge (Fig. S5D). We found less pulmonary accumulation of CD44^hi^CD4^+^ T cells from *Il2ra*^*-/-*^ donors than from WT donors but similar proportions of naïve CD44^lo^CD4^+^ T cells (Fig. S5E, F), indicating a known role for IL-2 signaling in T-cell expansion. Since IL-2Rα/CD25 neutralization 24 hours after initial sensitization had no significant impact on the number of T cells activated in the mLN (Fig. 5E, F) and recruited to the lungs (Fig. 5SA, B), this could indicate that IL-2 signaling within the first few hours after T-cell activation is most crucial for expansion. Nevertheless, *Il2ra*^*-/-*^ T cells had an impaired capacity to express Th2 cell cytokines even in low-IL-6 environments (Fig. S5G, H). However, they were perfectly able to produce IL-17 at levels comparable to those produced by WT T cells (Fig. S5I–J). Thus, inhibition of IL-2 signaling in allergen-stimulated T cells can prevent Th2 biasing in low-IL-6 environments or in T cells that do not respond to IL-6.

### The balance between IL-6 and IL-2 signals regulates the development of allergen-specific Th2 cell responses

We next decided to change the availability of IL-2 during early CD4^+^ T-cell activation. Thus, we treated WT:*Lck*^*cre*^*-Il6r*^*fl/fl*^ mixed BM chimeras with an anti-IL-2 mAb or rIL-2 during sensitization, followed by HDM challenge and lung analysis (Fig. 5I). In HDM^LPS^-sensitized mice, treatment with the anti-IL-2 mAb prevented Th2 cell cytokine production by *Il6r*^*-/-*^ CD44^hi^CD4^+^ T cells. In contrast, treatment with rIL-2 promoted Th2 cell cytokine production by WT CD44^hi^CD4^+^ T cells (Fig. 5J, K). Administration of the anti-IL-2 mAb or rIL-2 did not affect the capacity of T cells to produce IL-17 (Fig. S5K, L). These data showed that lung effector Th2 cell responses to HDM caused by a lack of IL-6 signaling could be prevented by neutralizing IL-2. In contrast, excess IL-2 signaling overcame the HDM^LPS^/IL-6-mediated suppression of Th2 cell responses.

We next evaluated whether the opposite effects of IL-2 and IL-6 on lung Th2 cell responses result from differential priming of antigen-specific CD4^+^ T cells after sensitization. First, we evaluated the effects of an anti-IL-2 mAb on donor 4get.OTII cell expansion and polarization (Fig. 5L). Anti-IL-2 treatment did not affect the expansion of donor OTII cells (Fig. 5M, N) but downregulated CD25 (Fig. S5M–O) and suppressed IL-4-GFP^+^ expression (Figs. 5M, O, P) in OTII cells from *Il6*^-/-^ recipients treated with HDM^LPS^. Thus, inhibition of IL-2 signaling by IL-2 neutralization prevented the Th2 cell polarization of antigen-specific T cells arising in the absence of IL-6.

Next, we evaluated the effect of administering rIL-2 on Th2 cell priming. These analyses were performed in combination with blockade of IL-6 signaling and T-bet deficiency, all of which contribute to enhanced Th2 cell responses. Specifically, we cotransferred WT (CD45.1^+^ CD45.2^+^) and Tbx21^−/−^ (CD45.2^+^) 4get.OT-II cells into CD45.1^+^ WT recipients, sensitized and treated the mice with anti-IL-6/IL-6R or rIL-2, and analyzed the expanded WT and *Tbx21*^*−/−*^ donor cells (Fig. 5Q). WT and *Tbx21*^*−/−*^ OTII cells accumulated similarly under all the conditions (Fig. 5R). For WT OTII cells from HDM^LPS^-treated mice, rIL-2 and anti-IL-6/IL-6R treatments enhanced the frequency of IL-4-GFP^+^ cells. The combination of both treatments did not have an additive effect. Similar results were observed for *Tbx21*^*−/−*^ OTII cells from HDM^LPS^-sensitized mice, although in this case, the absence of T-bet expression in the OTII cells had an additive effect with rIL-2 or anti-IL-6/IL-6R treatment. Overall, our data show that sustained IL-2 signaling after initial T-cell activation, which is particularly favored when T cells receive suboptimal IL-6 signaling, supports Th2 cell polarization of antigen-specific T cells via a mechanism independent of T-bet. Therefore, maintaining a proper balance between IL-6 and IL-2 appears to be crucial in controlling Th2 cell immunity to allergens.

### IL-6 prevents IL-2 responsiveness by inducing SOCS3 expression

**Fig. 6:**
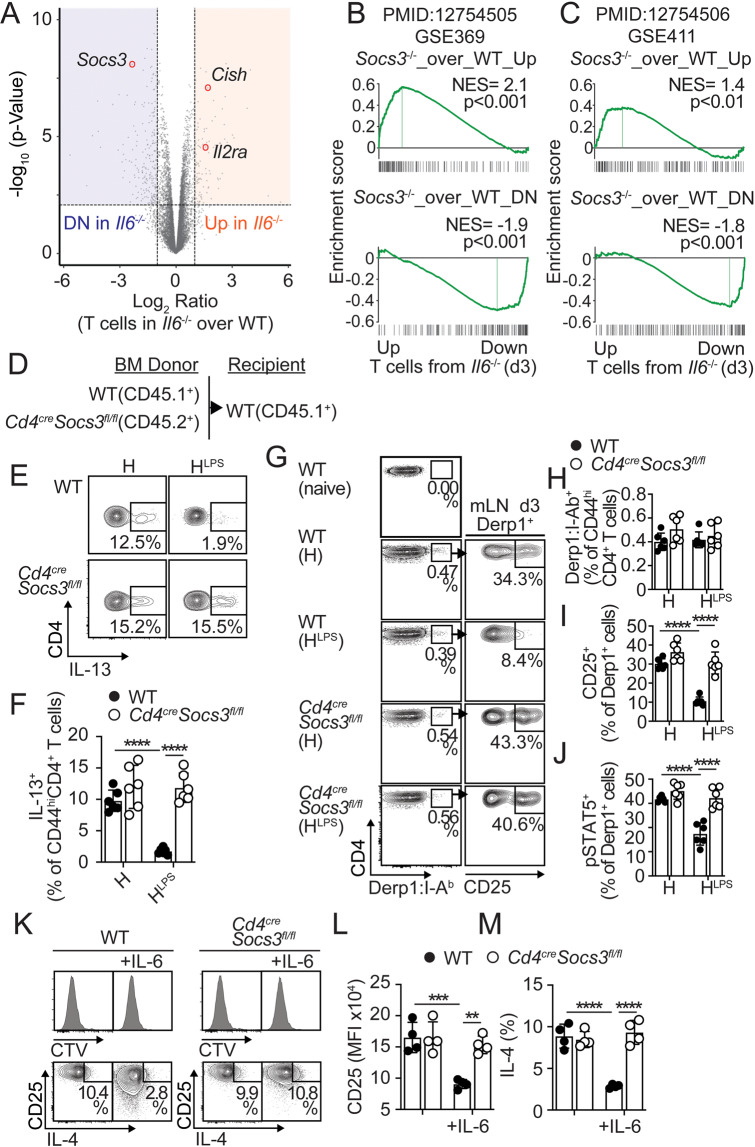
IL-6 suppresses allergen-specific Th2 cell responses by promoting SOCS3-mediated inhibition of IL-2 responsiveness. **A–C** WT and *Il6*^*-/-*^ mice were transferred with OTII cells and i.n. sensitized with HDM^LPS^ + OVA. On Day 3, OTII cells were sorted from the mLN, and RNA-seq was performed (three replicates). Volcano plot highlighting that the IL-2-driven genes *Il2ra* and *Cish* and the IL-6-driven gene *Socs3* were upregulated and downregulated in OTII cells from *Il6*^*-/-*^ mice, respectively (FDR < 0.05, ≥2 FC. See Table S1) (**A**). GSEA plots showing the enrichment of genes regulated by IL-6-driven SOCS3 in liver cells (**B**) and macrophages (**C**) in OTII cells from *Il6*^*-/-*^ versus WT mice. **D–F** WT:*CD4*^*cre*^*-Socs3*^*fl/fl*^ chimeras were i.n. sensitized with HDM or HDM^LPS^ and i.n. challenged with HDM. Frequencies of IL-13^+^ cells in the WT and *CD4*^*cre*^*-Socs3*^*fl/fl*^ CD44^hi^CD4^+^ T-cell compartments in the lungs (*n* = 6). **G–J** WT:*CD4*^*cre*^*-Socs3*^*fl/fl*^ chimeras were i.n. sensitized with HDM or HDM^LPS^ and analyzed on Day 3. Frequencies of total, CD25^+^, and pSTAT5^+^ Derp1-specific CD44^hi^CD4^+^ T cells in the WT and *CD4*^*cre*^*-Socs3*^*fl/fl*^ CD44^hi^CD4^+^ T-cell compartments in the mLN (*n* = 6). **K–M** CTV-labeled WT (CD45.1^+^) and *CD4*^*cre*^*-Socs3*^*fl/fl*^ (CD45.2^+^) CD4^+^ T cells from naïve mice were mixed 1:1 and stimulated in vitro with plate-bound anti-CD3 and soluble anti-CD28 for 48 hours. rIL-6 (1 ng/ml) or PBS was added for an additional 72 hours. CTV profiles (**K**), CD25 expression (**K, L**) and intracellular IL-4 expression in WT (**K, M**) and *CD4*^*cre*^*-Socs3*^*fl/fl*^ CD4^+^ T cells (values in quadruplicate). Data are representative of at least two independent experiments (mean±S.D., two-way ANOVA)

To better define the mechanism by which IL-6 controls IL-2 responsiveness in recently activated CD4^+^ T cells, we examined gene expression differences between OTII cells activated in IL-6–sufficient and IL-6–deficient environments (Table S1). As expected, on Day 3 after HDM^LPS^ sensitization, OTII cells primed in *Il6*^*−/−*^ mice overexpressed highly inducible IL-2 genes [25], such as *Il2ra* and *Cish* (Fig. 6A). In contrast, one of the genes most significantly downregulated was *Socs3* (Fig. 6A). In addition, genes previously identified to be regulated in IL-6-treated *Socs3*-deficient livers [30] (Fig. 6B) and macrophages [31] (Fig. 6C) were highly enriched in OTII cells from *Il6*^*−/−*^ mice. These data suggest dominant regulation by IL-6-induced SOCS3 in early T-cell activation in response to HDM^LPS^.

SOCS3 is strongly induced by IL-6 [32] and has been shown to prevent the activation of STAT3 by IL-6 [30, 31, 33]. However, SOCS3 can also inhibit signaling by many other cytokines that signal via the JAK/STAT pathway, including IL-12 [34], IL-10/TGFβ [35], IL-6/IL-23 [36], and IL-2 [37]. Importantly, loss of SOCS3 can result in exacerbated IFNγ [34] and IL-10/TGFβ [35] production by T cells that can indirectly suppress Th2 cell differentiation and effector function. To specifically test whether loss of SOCS3 expression intrinsically enhances IL-2 responsiveness in CD4^+^ T cells and Th2 cell polarization in response to HDM, we used WT:*CD4*^*cre*^*-Socs3*^*fl/fl*^ mixed BM chimeras (Fig. 6D). In these mice, WT and *Socs3*^*−/−*^ T cells coexisted in the same environment, avoiding indirect cytokine-mediated effects. We sensitized WT:*CD4*^*cre*^*-Socs3*^*fl/fl*^ mice with HDM or HDM^LPS^, challenged them with HDM, and then determined the frequency of IL-13^+^ Th2 cells within the WT and *Socs3*^−/−^ CD44^hi^CD4^+^ T-cell compartments. We found that while HDM^LPS^ sensitization prevented the accumulation of WT Th2 cells in the lungs, it failed to prevent the increase in the frequency of *Socs3*^−/−^ Th2 cells (Fig. 6E, F). These results indicated that SOCS3 expression in responding T cells was intrinsically required to suppress the Th2 cell differentiation program in response to HDM. Since Th2 cell commitment in response to HDM required strong IL-2 signaling, we next tested whether SOCS3 expression influences the IL-2 responsiveness of HDM-responsive T cells. Thus, we analyzed HDM/Derp1-specific T cells [38] in the mLNs of WT:*CD4*^*cre*^*-Socs3*^*fl/fl*^ mixed BM chimeras on Day 3 after HDM or HDM^LPS^ sensitization. The frequencies of Derp1-specific T cells within the WT and *Socs3*^−/−^ CD4^+^ T-cell compartments were similar after HDM or HDM^LPS^ sensitization (Fig. 6G, H). As expected, CD25 expression and IL-2-driven pSTAT5 were downregulated in WT Derp1-specific T cells from HDM^LPS^-treated mice compared with those from HDM-treated mice (Fig. 6G, I, J). In contrast, *Socs3*-deficient Derp1-specific T cells maintained elevated expression of CD25 and IL-2-driven pSTAT5 in HDM^LPS^-treated mice (Fig. 6G, I, J). These results show that SOCS3 expression in HDM-specific T cells is required to limit IL-2/STAT5 responsiveness shortly after T-cell activation. Since this effect was also dependent on IL-6 signaling, our results further suggest that IL-6 controls SOCS3 expression in T cells to constrain IL-2 responsiveness and Th2 cell commitment. To confirm this, CD3/CD28-stimulated WT and *Socs3*-deficient CD4^+^ T cells were cocultured in the presence of rIL-6. WT and *Socs3*-deficient CD4^+^ T cells proliferated at similar rates, either in the presence or absence of IL-6 (Fig. 6K). As expected, IL-6 reduced the expression of CD25 and IL-4 in WT CD4^+^ T cells, but it failed to exert a similar effect on *Socs3*-deficient CD4^+^ T cells (Fig. 6K–M). Overall, our findings suggest that IL-6-driven SOCS3 acts to inhibit IL-2 responses in recently activated T cells and subsequent Th2 cell lineage commitment.

### JAK1 inhibition blocks IL-2 signaling and prevents Th2 bias in the absence of IL-6

**Fig. 7:**
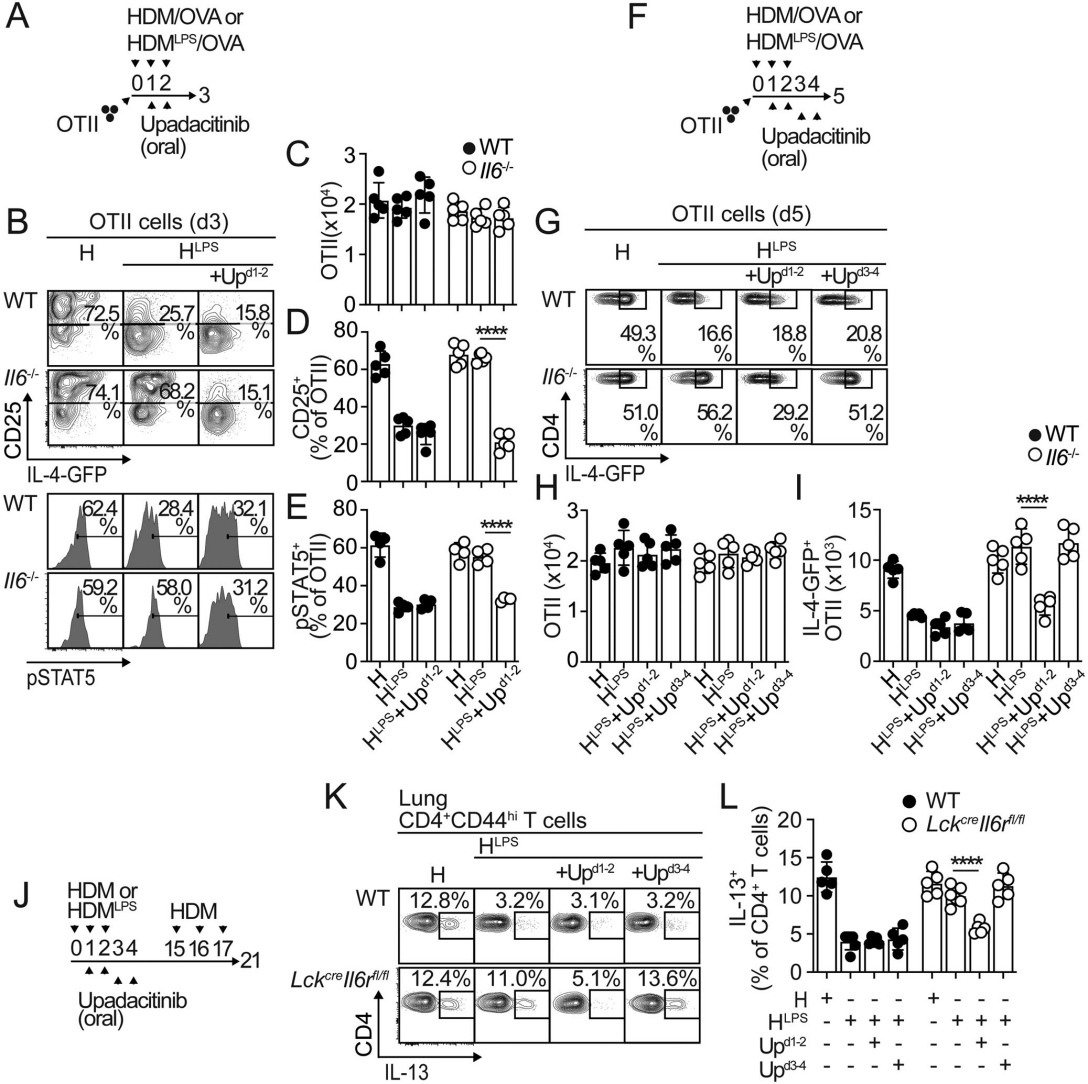
JAK1 inhibition prevents excessive IL-2 signaling and Th2 cell polarization in the absence of IL-6 signaling. **A–I** WT and *Il6*^*-/-*^ mice were transferred with OTII.4get cells, i.n. treated with HDM or HDM^LPS^ + OVA, orally treated with 20 mg/kg/day JAK1 inhibitor upadacitinib or vehicle at the indicated time points and analyzed on Day 3 (**A–E**) and Day 5 (**F–I**). Frequencies and numbers of total (**C, H**), CD25^+^ (**B, D**), pSTAT5^+^ (**B, E**), and IL-4-GFP^+^ (**G, I**) OTII cells in the mLN. (**J–L**) WT:*Lck*^*cre*^*-Il6r*^*fl/fl*^ chimeras were i.n. sensitized with HDM or HDM^LPS^, orally treated with upadacitinib or vehicle, and i.n. challenged with HDM (**J**). Frequencies of IL-13^+^ cells in the WT and *Lck*^*cre*^*-Il6r*^*fl/fl*^ CD44^hi^CD4^+^ T-cell compartments in the lungs (**K, L**). Data are representative of two independent experiments (mean±S.D., *n* = 5, one-way ANOVA)

JAK family members are key in initiating cytokine receptor signaling. IL-2 activates JAK1 and JAK3, with JAK1 playing a dominant role [39, 40]. Thus, we tested whether treatment of *Il6*^*-*/-^ mice with the JAK1 selective inhibitor upadacitinib blocks IL-2 signaling in recently activated T cells and hence their polarization to Th2 cells. Upadacitinib administered on Days 1 and 2 postsensitization (Fig. 7A) did not affect the expansion of donor OTII cells (Fig. 7C) but induced downregulation of CD25 expression (Fig. 7B, D) and IL-2-driven pSTAT5 (Figs. 7B and 7E) in donor OTII cells from HDM^LPS^-treated *Il6*^*-*/-^ mice. Furthermore, this course of upadacitinib treatment suppressed IL-4-GFP expression in donor OTII cells (Fig. 7G) and thus the accumulation of IL-4-GFP^+^4get.OTII cells (Fig. 7I) in HDM^LPS^-sensitized *Il6*^-/-^ recipients without affecting total OTII cell expansion (Fig. 7H). Nonetheless, later upadacitinib administration on Days 3 and 4 postsensitization did not affect IL-4-GFP expression in donor OTII cells (Fig. 7F–I). We further analyzed the effect of upadacitinib treatment on lung effector Th2 cell responses developed in the absence of IL-6 signaling. Thus, we treated WT:*Lck*^*cre*^*-Il6r*^*fl/fl*^ mixed BM chimeras (Fig. 7J) with upadacitinib at different times during sensitization (i.e., 1-2 vs. 3-4 days post sensitization) (Fig. 7K). The mice were then challenged, and cytokine production by WT and *Il6r*^*-/-*^ T cells was analyzed in the lungs. In HDM^LPS^-sensitized mice, treatment with upadacitinib on Days 1-2 prevented Th2 cell cytokine production by *Il6r*^*-/-*^ CD44^hi^CD4^+^ T cells, while treatment with upadacitinib on Days 3-4 had no effect on Th2 cell cytokine production by *Il6r*^*-/-*^ CD44^hi^CD4^+^ T cells (Fig. 7L, M). These data suggest that JAK1 inhibition in recently activated T cells can effectively suppress IL-2 signaling and subsequent Th2 cell lineage commitment.

Collectively, our data indicate that IL-6 signaling in allergen-specific T cells is required to prevent bias toward the Th2 cell subset and subsequent allergic inflammation. Our work supports a model in which IL-6 upregulates SOCS3 expression, which limits IL-2 signaling in antigen-specific T cells and thereby restricts the acquisition of a Th2 cell differentiation program. Furthermore, we found that upadacitinib, a JAK1 inhibitor, could suppress IL-2 signaling in recently activated T cells and their subsequent commitment to the Th2 cell lineage, suggesting that blocking the JAK1 pathway could be used to prevent new allergen sensitization in environments with reduced IL-6 signaling.

## Discussion

Defective IL-6 signaling caused by mutations in the IL-6 receptor complex [5–7] or downstream mediators, such as STAT3 [8–11], leads to pathological Th2-biased immune responses. Despite the negative role of aberrant Th2-biased responses in patients harboring these mutations, little is known about how Th2 immunity is favored in the context of defective IL-6 signaling. Our data demonstrate that defective IL-6 signaling in CD4^+^ T cells responding to allergens enables prolonged IL-2 signaling that favors Th2 cell differentiation. Thus, we show that IL-6 is key in limiting IL-2 responsiveness in activated CD4^+^ T cells and that by restricting IL-2 signaling, IL-6 suppresses the initiation of the Th2 cell differentiation program in these cells.

Our findings support a model in which IL-6 is produced in response to the detection of pathogen-associated molecular patterns (PAMPs) contained in allergens. Several observational studies have indicated that high levels of exposure to microbial products or PAMPs, particularly bacterial endotoxins or LPS, inversely correlate with the development of allergen-induced, Th2-driven diseases, such as allergic asthma and atopy [19–21, 41–43]. Moreover, polymorphisms that affect LPS signaling (i.e., polymorphisms in toll-like receptor 4 (TLR4) and CD14) are associated with altered susceptibility to allergen sensitization, particularly in high-LPS environments [44–51]. These data suggest that efficient detection of LPS is essential for suppressing Th2 cell immune responses to aeroallergens. In addition, airborne allergens can be contaminated with other microbial components besides LPS, such as derivatives of gram-positive bacteria or mycobacteria, and are likely to affect Th2 cell responses in a similar way to LPS [49, 52].

Although epidemiological studies support a pivotal role for LPS in suppressing the development of allergen-induced Th2 diseases, some studies in mouse models have shown that the activation of TLR4 by LPS or alternative ligands may sometimes be a mechanism for initiating Th2 immunity to inhaled allergens. Specifically, stimulation of TLR4, usually at low intensity, can convert suboptimal doses of allergens or inert proteins into immunogenic antigens that would not otherwise trigger an immune response. Mechanistically, the activation of the airway epithelium by TLR4 has been linked to the induction of Th2 cell responses by promoting the release of alarmin cytokines, which ultimately activates pro-Th2 functions in conventional type 2 dendritic cells (cDC2s) (reviewed in [43]). Conversely, LPS detection by inflammatory monocyte-derived dendritic cells (moDCs) has been linked to the suppression of Th2 immune responses to allergens [18, 43]. This pathway is favored by the induced production of granulocyte-macrophage colony-stimulating factor (GM-CSF) at pulmonary perivascular locations, which promotes the differentiation of newly recruited monocytes into moDCs that are well-equipped to detect LPS and other PAMPs and produce proinflammatory cytokines in response. If they are contaminated with LPS or other microbial components, protease allergens, particularly those with cysteine protease activity, such as HDM, have great potential to elicit GM-CSF responses and moDC differentiation and thus have the capacity to activate the mechanisms that suppress Th2 cell responses [18]. Mechanistically, tumor necrosis factor-alpha (TNFα) produced by moDCs instructs cDC2s to produce IL-12 [15, 18]. Consequently, CD4^+^ T cells interacting with cDC2s upregulate T-bet, precluding Th2 cell differentiation [14, 15, 53, 54] and subsequent pathogenic allergic responses [15]. However, our current data demonstrate that LPS detection can also prevent the development of allergic Th2 cell responses by an IL-12/T-bet-independent mechanism. Here, we found that LPS-driven IL-6 and subsequent signaling in allergen-activated CD4^+^ T cells contributed to preventing the Th2 cell differentiation program by restricting IL-2 signaling that would otherwise favor Th2 polarization. Therefore, IL-12/T-bet-dependent and IL-6-dependent pathways are separate mechanisms that work together to prevent abnormal Th2-biased immune reactions. Although both pathways might have overlapping roles in preventing Th2 allergic responses, a defect in either pathway leads to a lack of effective suppression of Th2 cell polarization and the development of allergic diseases. In particular, a defective IL-12/T-bet-dependent pathway operating during infancy could be responsible for the higher susceptibility to Th2 bias and the increased tendency to develop allergic airway inflammation observed in children [15]. Furthermore, human loss-of-function mutations affecting T-bet [55–57] or the IL-6-dependent signaling pathway [5–11] share the common feature of leading to pathological overactivation of Th2 immunity. Thus, both pathways must be active to ensure adequate prevention of Th2 cell responses in humans. However, our data support that when LPS levels or IL-12 production are high, allowing for strong T-bet-dependent signaling, the IL-6-dependent pathway is no longer necessary. Therefore, IL-6 most likely contributes to inhibiting Th2 cell commitment to antigens that elicit weak IL-12 responses, such as those provoking moderated TLR stimulation. This includes allergens with limited PAMP activity.

In addition, genetic background affects responsiveness to PAMPs. Our studies were performed in congenic B6 mice, which are highly sensitive to LPS compared to BALB/c mice. In this sense, different doses of LPS achieve suppressive effects on the B6 and BALB/c backgrounds. In any case, although BALB/c mice required more LPS to inhibit Th2 cell responses, the underlying mechanism was similarly dependent on IL-6 signaling. Therefore, specific genetic variation may affect LPS and PAMP sensitivity more generally; thus, it may affect the risk of developing allergic Th2 cell disease.

IL-2 is a well-known potent driver of T-cell expansion [58]. However, IL-2 has other immunomodulatory functions in antigen receptor-activated T cells. In particular, IL-2 can promote Th1 [59] and Th2 [27–29] fate decisions in proliferating CD4^+^ T cells while suppressing the differentiation of Th17 [59–61] and T follicular helper (Tfh) cells [62]. Th2 cells depend on IL-4 signaling and the transcriptional activities of STAT6 and GATA3 [63]. Thus, Th2 cells depend on the expression of the IL-4 receptor, which consists of IL4Rα and γ_c_. IL4Rα is not expressed by naïve CD4^+^ T cells and thus must be induced following antigen encounter to allow IL-4 responsiveness and potent Th2 cell differentiation. Studies indicate that IL-2 promotes Th2 fate decisions by inducing IL4Rα [29]. In addition, IL-2 stabilizes the accessibility of the *Il4* locus [27–29], allowing for early production of IL-4 [27, 28], which serves as a positive feedback loop that preserves the Th2 cell phenotype. The IL-2-driven induction of IL-4Rα and chromatin accessibility at the *Il4* locus require STAT5 [28, 29]. Thus, supporting data indicate a key role for IL-2-driven STAT5 activation in the priming of Th2 cell differentiation and in helping maintain this phenotype. On the other hand, our data support that IL-6 is a key negative regulator of Th2 cell differentiation by counteracting prolonged IL-2 signaling during priming. Other studies have shown that IL-6 promotes Th2 cell differentiation in vitro, most likely via an indirect effect resulting from the primary inhibition of IFNγ production [64]. IL-2 production and signaling are tightly regulated in vivo, whereas in vitro activation of T cells typically occurs under unrestricted IL-2 signaling. Thus, differences in IL-2 availability between in vivo and in vivo models may explain the opposing modulatory effects of IL-6 on Th2 cell differentiation in these two systems.

Th2 cytokines are produced by type-2 Tfh cells that remain in the lymph nodes and control IgE responses and by effector Th2 cells that migrate to the lungs and promote eosinophilic airway inflammation. IL-6 was associated with the inhibition of Th2 cytokine production soon after initial T-cell activation and proliferation. Additionally, IL-6 was linked to the suppression of airway eosinophilia and specific IgE responses. Thus, our study supports a role for early IL-6 signaling in inhibiting the initial commitment of T cells to the Th2 cell cytokine production that precedes Tfh and effector T cell differentiation.

IL-2 binds with high affinity to a cell-surface receptor complex consisting of IL-2Rα/CD25, IL-2Rβ/CD122, and γ_c_. IL-2 can also bind with low affinity to the IL-2Rβ/CD122 and γ_c_ dimer. IL-2 induces the expression of IL-2Rα/CD25 [25], creating a positive feedback loop. We found that IL-6 interrupted this positive feedback loop, causing cessation of IL-2Rα/CD25-induced expression and thus rendering activated CD4^+^ T cells unable to respond to IL-2 with high affinity. The tyrosine-protein kinases JAK1 and JAK3, which are associated with the IL-2Rβ/CD122 and γ_c_ subunits, respectively, are activated after IL-2 binding. This creates docking sites for STAT5 that facilitate STAT5 activation and subsequent nuclear translocation to allow transcription of target genes [65]. SOCS family proteins are negative feedback inhibitors of cytokine-driven signaling that act through the JAK/STAT pathway [3]. In particular, studies have identified SOCS1 [66] and SOCS3 [37] as inhibitors of IL-2 signaling, both of which function by inhibiting the kinase activity of JAK1. We demonstrate that SOCS3 is strongly induced by IL-6 in antigen receptor-activated CD4^+^ T cells and potently inhibits IL-2-induced STAT5 activation and Th2 cell differentiation in response to HDM allergens. In contrast, other studies have shown that SOCS3 expression in T cells can indirectly promote Th2 cell responses by suppressing the upregulation of IFNγ [34], IL-10, or TGFβ [35] production. Since SOCS3 can inhibit multiple cytokine-mediated JAK/STAT signaling pathways, the more dominant effect depends on which particular cytokine pathway dominates the signaling in T cells. In our model, the increase in IL-2 signaling in the absence of SOCS3 promoted Th2 cell commitment and outweighed any effects that might have resulted from possible upregulation of IL-12/IFNγ signaling.

Mimicking the SOCS3-mediated inhibition of IL-2 signaling, we further showed that a selective JAK1 inhibitor could inhibit IL-2-driven STAT5 activation and polarization toward a Th2 phenotype, particularly in IL-6-deficient environments that promoted reduced SOCS3 activity in activated CD4^+^ T cells and presumably high JAK1 activation. JAK1 has also been implicated as a transducer of IL-4 signaling [67]. Thus, JAK1 inhibition could directly inhibit Th2 cell differentiation by interfering with an IL-4-driven positive feedback loop. However, our data showed that at doses that inhibited IL-2-driven STAT5 activation, the JAK1 inhibitor did not markedly alter the IL-4 signaling system. In brief, our data reveal a major role for JAK1 in IL-2-mediated signaling and Th2 bias that could have therapeutic implications.

Our results show that IL-6 interferes with high-affinity IL-2 signaling, which is critically required for Th2 fate commitment. However, IL-6 signaling can also inhibit the upregulation of IL-2Rβ/CD122, thereby inhibiting low-affinity IL-2 signaling [68]. This mechanism has been shown to control a state of persistent IL-2 hyporesponsiveness upon late and sustained antigen receptor triggering that enables the generation of Tfh cells in the germinal center (GC) [68]. Without this mechanism of IL-6-driven inhibition of IL-2 signaling, Tfh cells undergoing cognate interactions with GC B cells initiate an IL-2 signaling cycle that inhibits the maintenance of their Tfh cell phenotype. Our results, together with the aforementioned data, demonstrate that IL-6 can control IL-2 signaling by different mechanisms and at various T-cell activation and differentiation stages, which determine particular immunomodulatory functions. However, since IL-2 signaling is primarily initiated by T-cell receptor activation, it stands to reason that the role of IL-6 in counteracting IL-2 signaling is more prominent upon ongoing antigen presentation, such as at the onset of the immune response or in the GC. Conventional dendritic cells and B cells act as antigen-presenting cells in these situations, so future experiments should determine whether these cells are a source of IL-6, as previously suggested [69, 70], and identify the specific pathways that determine IL-6 production.

Taken together, our data demonstrate that IL-6 signaling in allergen-specific T cells is essential for preventing Th2 development by counteracting IL-2-driven pro-Th2 signals. This action of IL-6 on allergen-responsive T cells is mediated by upregulation of SOCS3 and could be mimicked by pharmacological inhibition of JAK1. Our data provide insights into the immunological processes behind skewed Th2 responses in patients with defective IL-6 signaling and have the potential to lead to new therapeutic options for these patients.

## Materials and methods

### Mouse strains

The mouse strains used in these experiments included C57BL/6 J (B6), B6.SJL-Ptprca Pepcb/BoyJ (CD45.1^+^ B6 congenic), C57BL/6-Tg(TcraTcrb)425Cbn/J (OTII), B6.129-Il4tm1Lky/J (B6.4get IL-4 reporter mice), B6.129S6-Tbx21tm1Glm/J (*Tbx21*^*−/−*^), B6.129S2-IL-6tm1Kopf/J (*Il6*^*−/−*^), B6.129S4-Il2ratm1Dw/J (*Cd25*^*−/−*^), B6.Cg-Tg(Lck-cre)3779Nik/J (*Lck-cre*), B6;SJL-Il6ratm1.1Drew/J (*Il6ra*^*fl/fl*^), B6N.129-Il21rtm1Kopf/J (*IL-21r*^*−/−*^), B6.Cg-Tg(Cd4-cre)1Cwi/BfluJ (*Cd4-cre*), and B6;129S4-Socs3tm1Ayos/J (*Socs3*^*fl/fl*^). B6.4get mice were originally obtained from Dr. M. Mohrs (Trudeau Institute). All other mice were originally obtained from The Jackson Laboratory (Bar Harbor, ME, U.S.) and were bred and housed at the University of Alabama at Birmingham animal facility under specific pathogen–free conditions. Experiments were performed with equal numbers of male and female mice. The University of Alabama at Birmingham Institutional Animal Care and Use Committee approved all procedures involving animals.

### Immunizations

HDM (*Dermatophagoides pteronyssinus* and *D. farina*) extract (<30 EU/mg endotoxin) was obtained from Greer Laboratories (Lenoir, NC, U.S.). Endotoxin quantification was performed using the Pierce Chromogenic Endotoxin Quant Kit (A39552S; Thermo Fisher Scientific). Mice were intranasally administered (i.n.) 100 µg of HDM extract, +/- 5 µg of LPS-free EndoFit OVA ( < 0.1 EU/mg endotoxin; InvivoGen, San Diego, CA, U.S.) +/- LPS from *Escherichia coli* 0111:B4 (Sigma‒Aldrich, Saint Louis, MO, U.S.) +/- 100 ng of rIL-6 (PeproTech, Cranberry, NJ, U.S.) daily for 3 days and challenged (i.n.) with 100 µg of HDM + /− 5 µg of LPS-free EndoFit OVA for 3 days. The i.n. administrations were given in 100 µl of PBS. In some experiments, mice were intraperitoneally administered (i.p.) 250 μg of a mix of anti–IL-6/R (15A7; BioXCell, Lebanon, NH, U.S.) and anti-IL-6 (MP5-20F3; BioXCell) neutralizing Abs, 250 µg of anti-CD25 neutralizing Ab (PC-61.5.3; BioXCell), 250 μg of a mix of anti-IL-2 neutralizing Abs (JES6-1A12 and S4B6-1; BioXCell), or 60,000 U rIL-2 (National Cancer Institute, Bethesda, MD, U.S.) at the indicated time points. In some experiments, mice were orally treated with the JAK1 inhibitor upadacitinib (ABT-494; Selleck Chemicals, Radnor, PA, U.S.) at a dose of 20 mg/kg per day in palm oil at the indicated time points.

### BM chimeras

Recipient mice were irradiated with 950 Rads from a high-energy X-ray source delivered in a split dose and reconstituted with 10^7^ total BM cells. Mice were allowed to reconstitute for at least 8–12 weeks before HDM treatment.

### Cell preparation and flow cytometry

Lungs were isolated, cut into small fragments and digested for 45 min at 37 °C with 0.6 mg/ml collagenase A (Sigma‒Aldrich) and 30 μg/ml DNase I (Sigma‒Aldrich) in RPMI-1640 medium (Thermo Fisher Scientific). Digested lungs, mLNs and spleens were mechanically disrupted by passage through a wire mesh. Red blood cells were lysed with 150 mM NH4Cl, 10 mM KHCO3 and 0.1 mM EDTA. Fc receptors were blocked with anti-mouse CD16/32 (5 μg/ml; BioXCell), followed by staining with fluorochrome-conjugated Abs. Fluorochrome-labeled anti-B220 (RA3-6B2), anti-CD3 (17A2), anti-CD4 (GK1.5), anti-CD11b (M1/70), anti-CD11c (HL3), anti-CD25 (PC61), anti-CD44 (IM7), anti-CD45.1 (A20), anti-CD45.2 (104), anti-CD138 (281-2), anti-IgE (R35-72y) anti-Ly6C (AL-21), anti-Ly6G (IA8), and anti-Siglec-F (E50-2440) were purchased from BD Biosciences (San Jose, CA, U.S). The I-A^b^ HDM Derp1_217-227_ MHC class II tetramer was obtained from the NIH Tetramer Core Facility (Atlanta, GA, U.S.). Dead cell exclusion was performed using 7-AAD (Calbiochem, San Diego, CA, U.S.). For intracellular cytokine staining of T cells, cell suspensions were stimulated with PMA (20 ng/ml) plus Calcimycin (1 μg/ml) in the presence of BD GolgiPlug for 5 h. The restimulated cells were surface stained, fixed and permeabilized with the BD Cytofix/Cytoperm Plus Kit, and stained with antibodies against IL-13 (13 A; eBioscience, San Diego, CA, U.S.), IL-5 (TRFK5; BioLegend, San Diego, CA, U.S.), IL-4 (11B11; BD Biosciences), IFNγ (XMG1.2; BD Biosciences), and IL-17 (TC11-18H10.1; BioLegend). For pSTAT5 staining of ex vivo CD4^+^ T-cell populations, cell suspensions were stimulated with 1 µg/ml rIL-2 for 15 min and then fixed and permeabilized with BD Cytofix/Cytoperm buffer (BD) and Phosflow Perm Buffer III (BD), followed by washing with Transcription Factor Phospho Perm Wash Buffer (BD) and incubation with anti-pSTAT5 pY694 (47; BD Biosciences). For pSTAT5 staining of in vitro-activated CD4^+^ T cells, cells were fixed, permeabilized, and stained without stimulation with rIL-2. Foxp3 intracellular staining was performed using a Mouse regulatory T-cell staining kit (eBioscience) and Abs against Foxp3 (FJK-16s; eBioscience). Flow cytometry was performed on an Attune NxT instrument. Cells were gated as viable (7AAD^-^) CD4^+^ T cells (B220^-^CD3^+^CD4^+^), congenic CD44^hi^CD4^+^ T cells (B220^-^CD3^+^CD4^+^CD44^hi^ followed by CD45.1^+^ or CD45.2^+^ gating), eosinophils (autofluorescence^-^Siglec-F^+^, these cells were additionally CD11b^+^F4/80^+^CD11c^lo^CX3CR1^-^CD64^-^), congenic OTII cells (B220^-^CD3^+^CD4^+^CD44^hi^ followed by CD45.1^+^ and/or CD45.2^+^ gating) and Derp1-specific T cells (I-A^b^ HDM Derp1_217-227_^+^_,_ B220^-^CD3^+^CD4^+^CD44^hi^). Gate-positive populations were identified by using fluoresce minus one (FMO) controls. I-A(d) CLIP_87-101_ staining was used as a negative control for I-A^b^ HDM Derp1_217-227_ tetramer staining.

### Cell purifications, sorting, cell transfers and in vitro cultures

CD4^+^ T cells were isolated from the spleen of naïve mice by MACS (Miltenyi Biotec, Gaithersburg, MD, U.S.). Equivalent numbers (5 × 10^4^–5 × 10^5^) of naïve OTII cells were transferred (i.v.) into naïve congenic recipients. For some experiments, transferred donor OTII cells were sorted from recipient mice after staining with fluorochrome-conjugated anti-B220, anti-CD4, anti-CD44, and anti-CD45.1. All sorting experiments were performed using a FACSAria (BD Biosciences) sorter at the University of Alabama at Birmingham Flow Cytometry core. Sorted cells were more than 98% pure, as determined by flow cytometry. For in vitro cultures, CD4^+^ T cells were labeled for 10 min at 37 °C with CellTrace Violet CTV (Molecular Probes, Thermo Fisher Scientific) and then activated with plate-bound anti-CD3 (clone 145-2C11; 1.5 μg/ml) and soluble anti-CD28 (clone 37.51; 1.5 μg/ml) Abs for 48 hours at 37 °C in flat-bottom 96-well plates. The indicated concentrations of an anti-IL-2 neutralizing Ab (JES6-1A12 and S4B6-1; BioXCell), an anti-CD25 neutralizing Ab (PC-61.5.3; BioXCell) and rIL-6 (PeproTech) were added for an additional 72 hours. Complete medium comprised RPMI 1640 medium supplemented with sodium pyruvate, HEPES (pH range, 7.2 to 7.6), nonessential amino acids, penicillin, streptomycin, 2-mercaptoethanol and 10% heat-inactivated FBS (all from Gibco, Thermo Fisher Scientific).

### BALF collection and measurement of cytokines

BALF was collected using 0.5–1 ml of sterilized PBS per mouse. The BALF samples were centrifuged at 5000 × *g* for 10 min, and the supernatants were frozen at −80 °C. IL-6 measurement was performed using the IL-6 Mouse Uncoated ELISA Kit (88-7064-22; Invitrogen, Waltham, MA, U.S.).

### HDM-specific IgE antibody measurement

Ninety-six-well plates (Corning Clear Polystyrene 96-Well Microplates) were coated overnight with HDM extracts at 200 μg/ml in 0.05 M Na2CO3 pH 9.6. The coated plates were then blocked for 1 h with 1% BSA in PBS. Serum from mice was collected and serially diluted (threefold) in PBS with 10 mg/ml BSA and 0.1% Tween 20 before incubation in the coated plates. After washing, bound antibody was detected with HRP-conjugated goat anti-mouse IgE (1:2,000, 1110-05; Southern Biotech, Birmingham, AL, U.S.) and quantified by spectrophotometry at 405 nm (OD).

### RNA sequencing (RNA-seq)

#### Primary analysis

Library preparation and RNA-seq were conducted through Genewiz (South Plainfield, NJ, U.S.). Libraries were sequenced using a 1 × 50–base pair single-end rapid run on the HiSeq 2500 platform. The quality of raw sequence fastq-formatted files was assessed using fastQC (http://www.bioinformatics.babraham.ac.uk/projects/fastqc). Sequences were trimmed using Trim Galore (version 0.4.4) with phred33 scores, paired-end reads and the Nextera adapter options (http://www.bioinformatics.babraham.ac.uk/projects/trim_galore). Trimmed sequences were aligned using STAR (version 2.5.2a) with mouse GRCm39 and default options [71]. Aligned reads were counted with the HTseq-count (version 0.6.1p1) set for unstranded reads using the GRCm39 annotation file [72].

#### Downstream analysis

The R package edgeR [73] was used to assess differential expression between groups and to generate gene-by-sample matrices. A volcano plot was generated using R. Comparison of our data to other published datasets was accomplished using gene set enrichment analysis (GSEA) [74]. Upstream regulator analysis was performed using Ingenuity Pathway Analysis (IPA; Qiagen Digital Insights, Redwood City, CA, U.S.) [75].

### Quantification and Statistical analysis

All plots and histograms were plotted in FlowJo v.9 and v.10 software (TreeStar). GraphPad Prism (Version 9) was used for data analysis. The statistical significance of differences in mean values was determined using a two-tailed Student’s *t* test or one-way/two-way ANOVA with post hoc Tukey’s multiple comparison test. *P* values of less than 0.05 were considered statistically significant (^*^P < 0.05, ^**^P < 0.01, ^***^P < 0.001, ^****^P < 0.0001).

## Supplementary information

Supplementary Figures

Table S1

Table S2

## Data Availability

The raw data fastq files, processed data count files and a counts per million table for the RNA-seq analyses reported in this paper have been deposited in the GEO database under accession code GSE212158. This paper does not report the original code.
