# Supplementary material for: IL-6 prevents Th2 cell polarization by promoting SOCS3-dependent suppression of IL-2 signaling
Source: Cell Mol Immunol. 2023 Apr 12;20(6):651–65. doi: 10.1038/s41423-023-01012-1 (PMC10229632; doi:10.1038/s41423-023-01012-1)
Supplement: Supplementary file 1 — Supplementary Figures [file 41423_2023_1012_MOESM1_ESM.docx]

**SUPPLEMENTARY MATERIALS**

Fig. S1. IL-6 signaling during allergen sensitization oppositely regulates Th2 and Th17 cell responses to HDM.

Fig. S2. IL-21 signaling does not affect allergen-specific Th2 cell-mediated immunity.

Fig. S3. IL-6 signaling is not required to suppress Th2 cell differentiation in the presence of high LPS or IL-12.

Fig. S4. IL-6 signaling in responder T cells prevents prolonged IL-2 responsiveness.

Fig. S5. IL-2 signaling on allergen-specific T cells does not regulate polarization toward a Th17 profile.

Table S1. Raw data file (Excel)

Table S2. Raw data file (Excel)

**
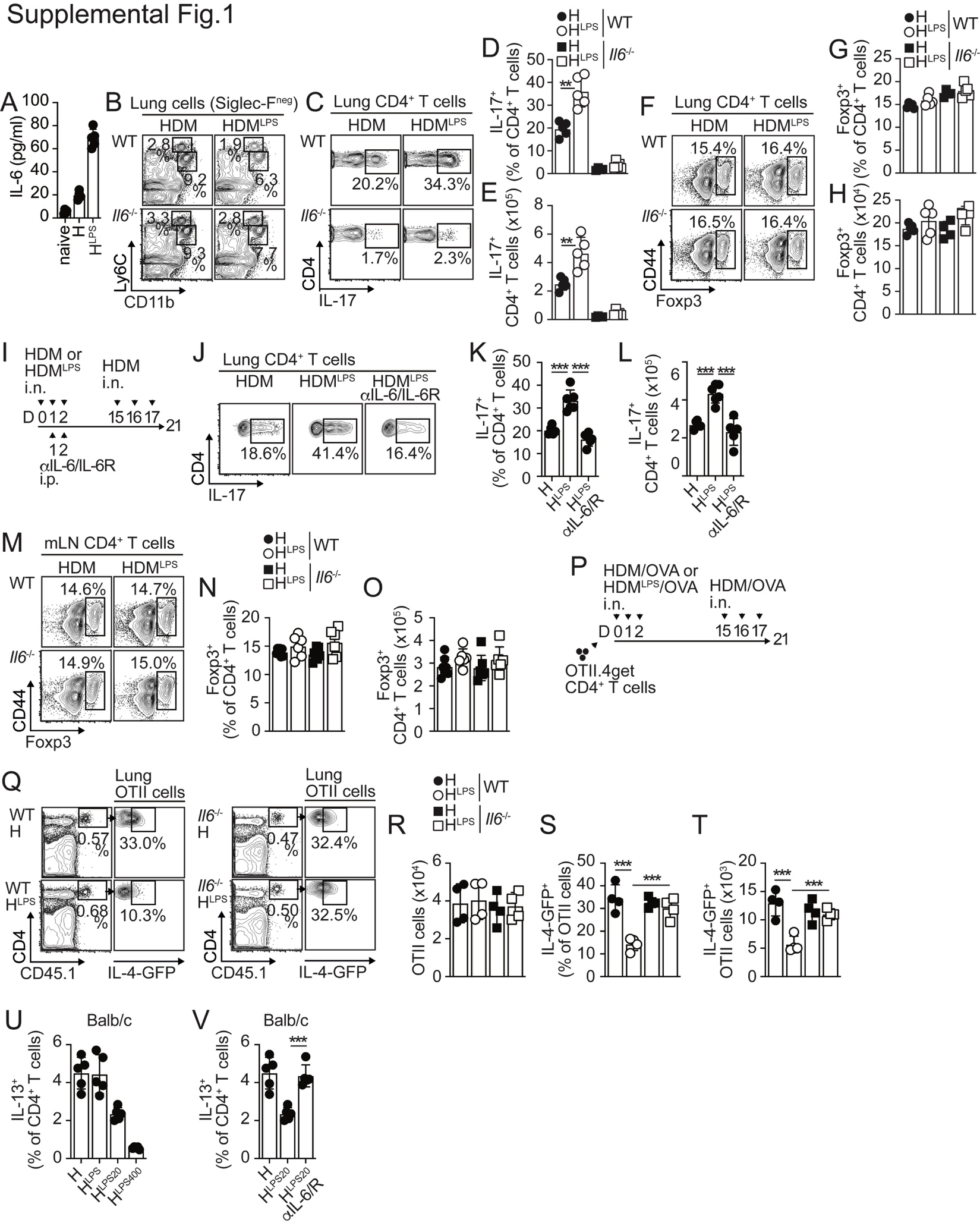
**

**Fig. S1. IL-6 signaling during allergen sensitization oppositely regulates Th2 and Th17 cell responses to HDM.**

(**A**) IL-6 in BAL from untreated or HDM- or HDM^LPS^-treated B6 mice analyzed on day 1. (**B-H**) B6 (WT) and *Il6^-/-^* mice were i.n. sensitized with HDM or HDM^LPS^ and challenged with HDM. Frequencies of CD11b^int^Ly6C^hi^ monocytes and CD11b^hi^Ly6C^int^ neutrophils in the lungs (**B**). Frequencies (**C-D**) and numbers (**E**) of IL-17^+^ CD4^+^ T cells in the lungs. Frequencies (**F-G**) and numbers (**H**) of Foxp3^+^ CD4^+^ T cells in the lungs. (**I-L**) B6 mice were i.n. sensitized with HDM or HDM^LPS^. Some mice also received 250µg anti-IL-6 and anti-IL-6R (i.p.). On day 15, mice were i.n challenged with HDM and analyzed on day 21 (**I**). Frequencies (**J-K**) and numbers (**L**) of IL-17^+^ CD4^+^ T cells in the lungs. (**M-O**) Frequencies (**M-N**) and numbers (**O**) of Foxp3^+^ CD4^+^ T cells in the mLNs on day 5 after sensitization. (**P-T**) Mice were transferred with OTII.4get cells, i.n sensitized with HDM or HDM^LPS^ + OVA, and challenged with HDM+OVA (**P**). Frequencies and numbers of total (**Q-R**) and IL-4-GFP^+^ (**Q, S-T**) OTII cells in the lung. (**U-V**) BALB/c mice were i.n sensitized with 100μg HDM containing different amount of LPS (HDM^LPS^: 1μg LPS/mg, HDM^LPS20^: 20μg LPS/mg, HDM^LPS400^: 400μg LPS/mg). Some mice also received 250µg anti-IL-6 and anti-IL-6R (i.p.). Mice were then challenged with HDM. Frequencies of IL-13^+^ CD4^+^ T cells in the lungs. Data are representative of at least three independent experiments (mean±S.D., n=4-6, two-way and one-way Anova).


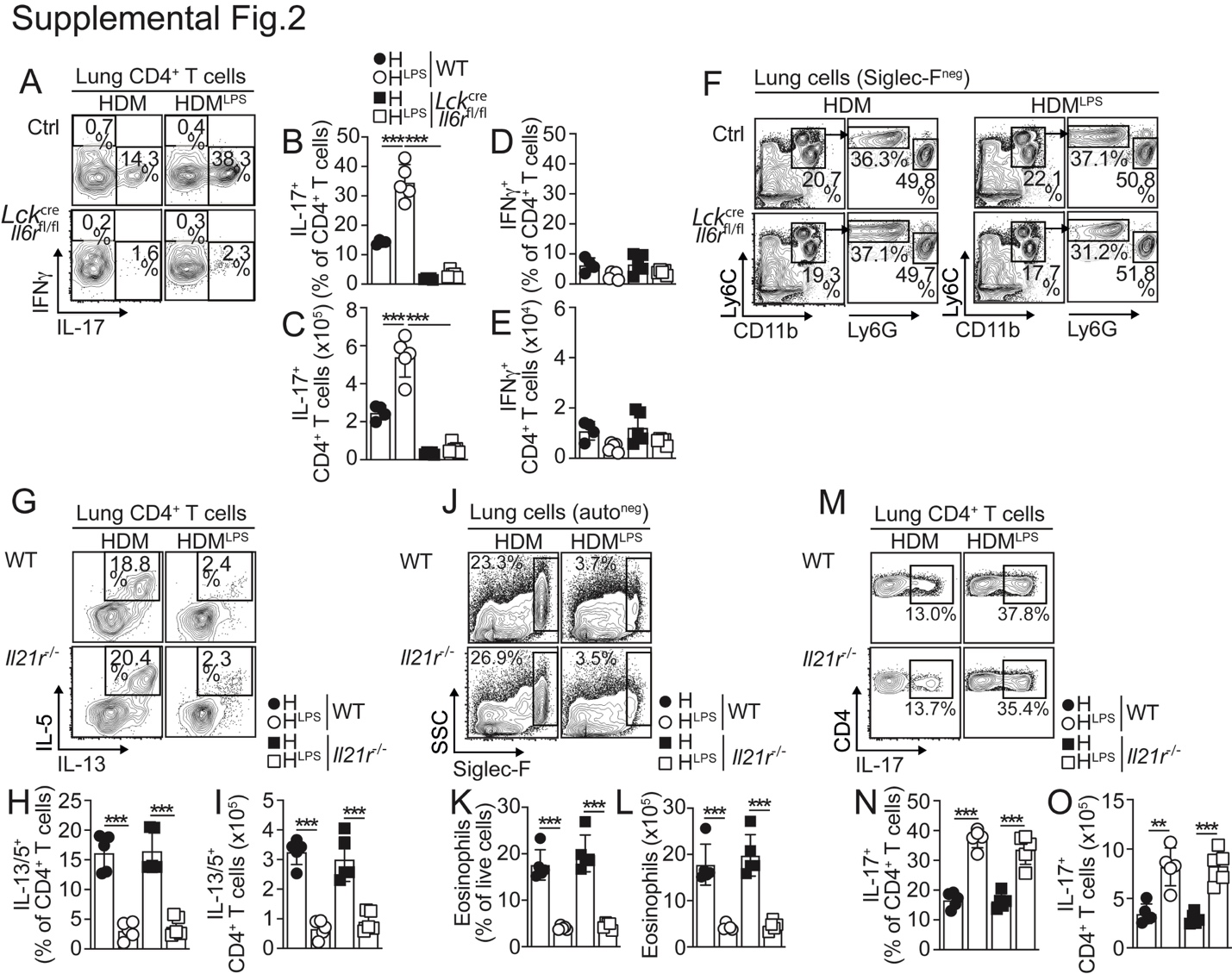


**Fig. S2. IL-21 signaling does not affect allergen-specific Th2 cell-mediated immunity.**

(**A-F**) *Lck^cre^-Il6r^fl/fl^* and control mice were i.n. sensitized with HDM or HDM^LPS^ and challenged with HDM. Frequencies and numbers of IL-17^+^ (**A-C**) and IFNγ^+^ (**A, D-E**) CD4^+^ T cells in the lung. Frequencies of CD11b^int^Ly6C^hi^Ly6G^lo^ monocytes and CD11b^hi^Ly6C^int^Ly6G^hi^ neutrophils in the lungs (**F**). (**G-O**) B6 (WT) and *Il21r^-/-^* mice were i.n. sensitized with HDM or HDM^LPS^ and challenged with HDM. Frequencies (**G-H**) and numbers (**I**) of IL-13^+^IL-5^+^ CD4^+^ T cells in the lungs. Frequencies (**J-K**) and numbers (**L**) of eosinophils in the lungs. Frequencies (**M-N**) and numbers (**O**) of IL-17^+^ CD4^+^ T cells in the lungs. Data are representative of two independent experiments (mean±S.D., n=4-5, two-way Anova).


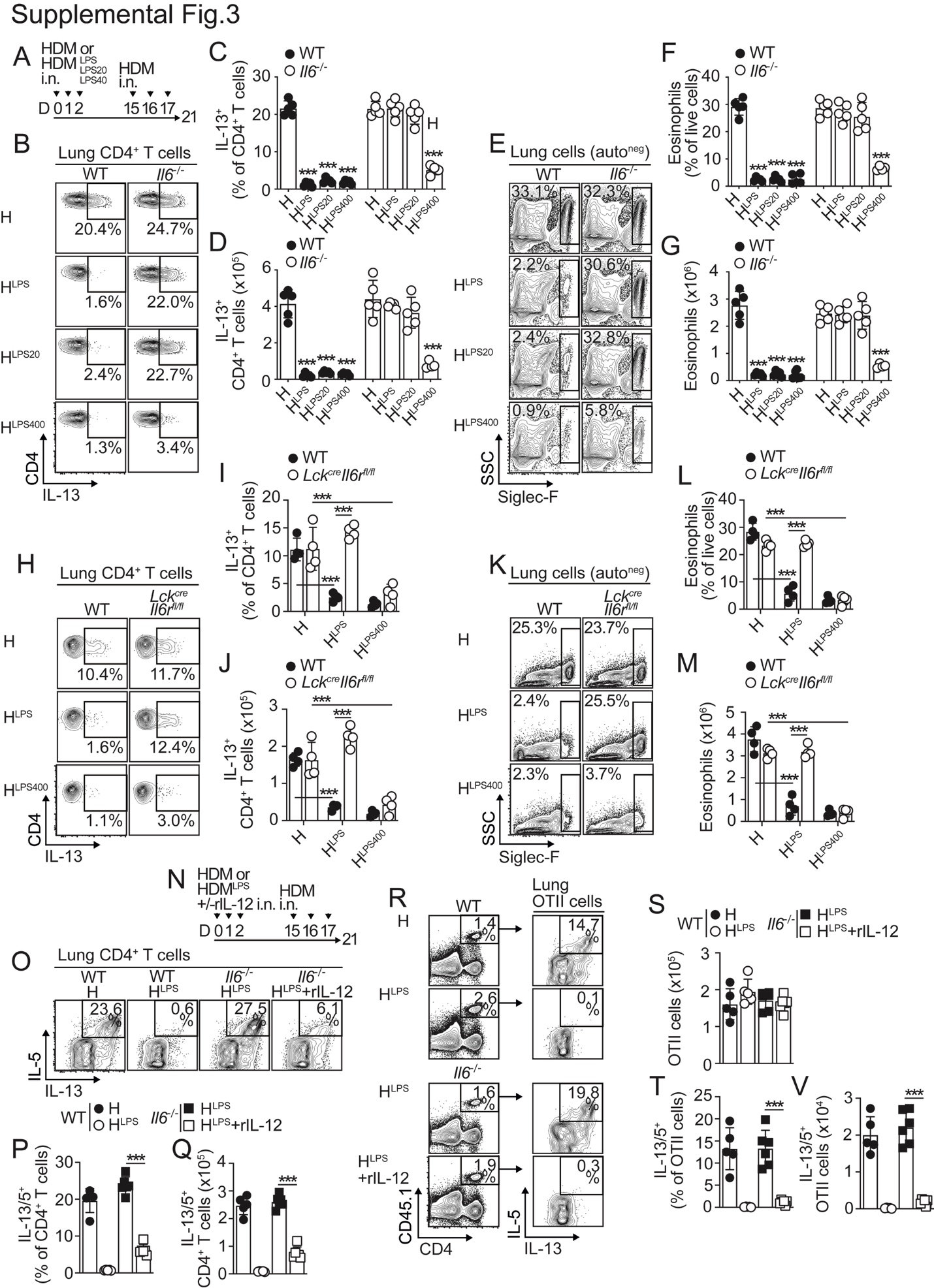


**Fig. S3. IL-6 signaling is not required to suppress Th2 cell differentiation in the presence of high LPS or IL-12.**

(**A-G**) WT and *Il6^-/-^* mice were i.n sensitized with 100μg HDM containing different amount of LPS (HDM^LPS^: 1μg LPS/mg, HDM^LPS20^: 20μg LPS/mg, HDM^LPS400^: 400μg LPS/mg). Mice were then challenged with HDM (**A**). Frequencies (**B-C**) and numbers (**D**) of IL-13^+^ CD4^+^ T cells in the lungs. Frequencies (**E-F**) and numbers (**G**) of eosinophils in the lungs. (**H-M**) *Lck^cre^-Il6r^fl/fl^* and control mice were i.n. sensitized with containing different amount of LPS (HDM^LPS^: 1μg LPS/mg, HDM^LPS400^: 400μg LPS/mg) and challenged with HDM (**A**). Frequencies (**H-I**) and numbers (**J**) of IL-13^+^ CD4^+^ T cells in the lungs. Frequencies (**K-L**) and numbers (**M**) of eosinophils in the lungs. (**N-Q**) WT and *Il6^-/-^* mice were i.n sensitized with HDM or HDM^LPS^+/-150ng rIL-12 and challenged with HDM (**N**). Frequencies (**O-P**) and numbers (**Q**) of IL-13^+^IL-5^+^ CD4^+^ T cells in the lungs. (**R-V**) WT and *Il6^-/-^* mice were transferred with OTII.4get cells, i.n sensitized with HDM+OVA or HDM^LPS^+OVA+/-150ng rIL-12, and i.n. challenged with HDM+OVA. Frequencies and numbers of total (**R-S**) and IL-13^+^IL-5^+^ (**R, T-V**) OTII cells in the lung. Data are representative of two independent experiments (mean±S.D., n=4-5, one-way Anova).


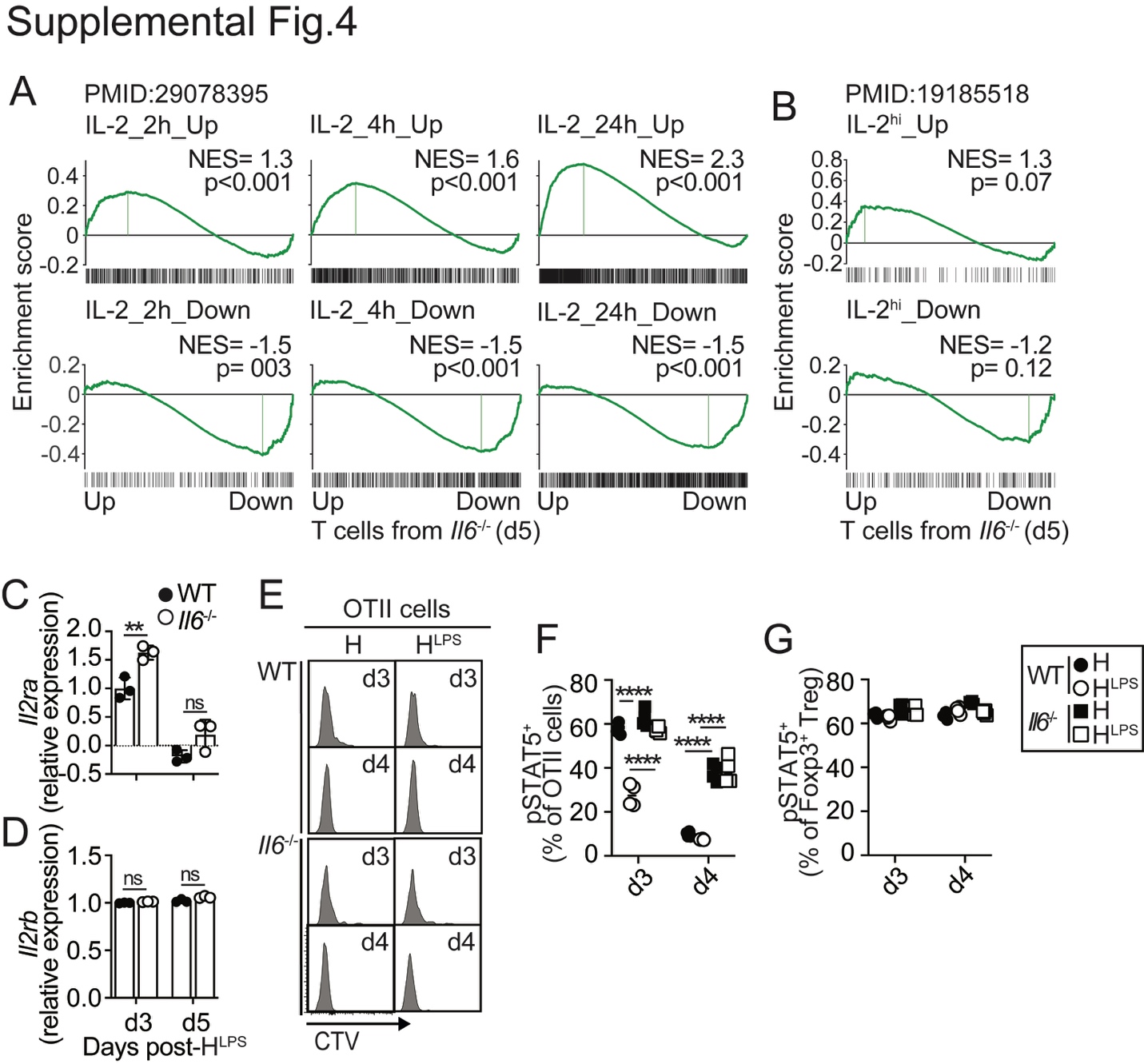


**Fig. S4. IL-6 signaling in responder T cells prevents prolonged IL-2 responsiveness.**

(**A-B**) WT and *Il6^-/-^* mice were transferred with OTII cells and i.n sensitized with HDM^LPS^+OVA. On day 5, OTII cells were sorted from mLN and RNA-seq was performed (three replicates). 156 differentially expressed genes, with 43 up- and 113 down-regulated in OTII from *Il6^-/-^* mice, were identified (FDR <0.05, ≥2 FC. See **Table S1**). GSEA plots showing the enrichment of genes in OTII cells from *Il6^-/-^* versus WT mice for genes regulated by IL-2 (**A**) or by strong IL-2 signaling (**B**). (**C-D**) Expression of *Il2ra* (**C**) and *Il2rb* (**D**) in donor OTII cells from HDM^LPS^-treated WT and *Il6^-/-^* mice on days 3 and 5. (**E-G**) WT and *Il6^-/-^* mice were transferred with CTV-labeled OTII.4get cells and i.n sensitized with HDM or HDM^LPS^ + OVA. CTV profiles in donor OTII cells from mLNs (**E**). Cells from mLNs were stimulated with 1ug/ml rIL-2 for 15 min and STAT5 phosphorylation in OTII (**F**) and Foxp3^+^ CD4^+^ T cells (**G**) was determined. Data are representative of two independent experiments (mean±S.D., n=3-4, two-way Anova).


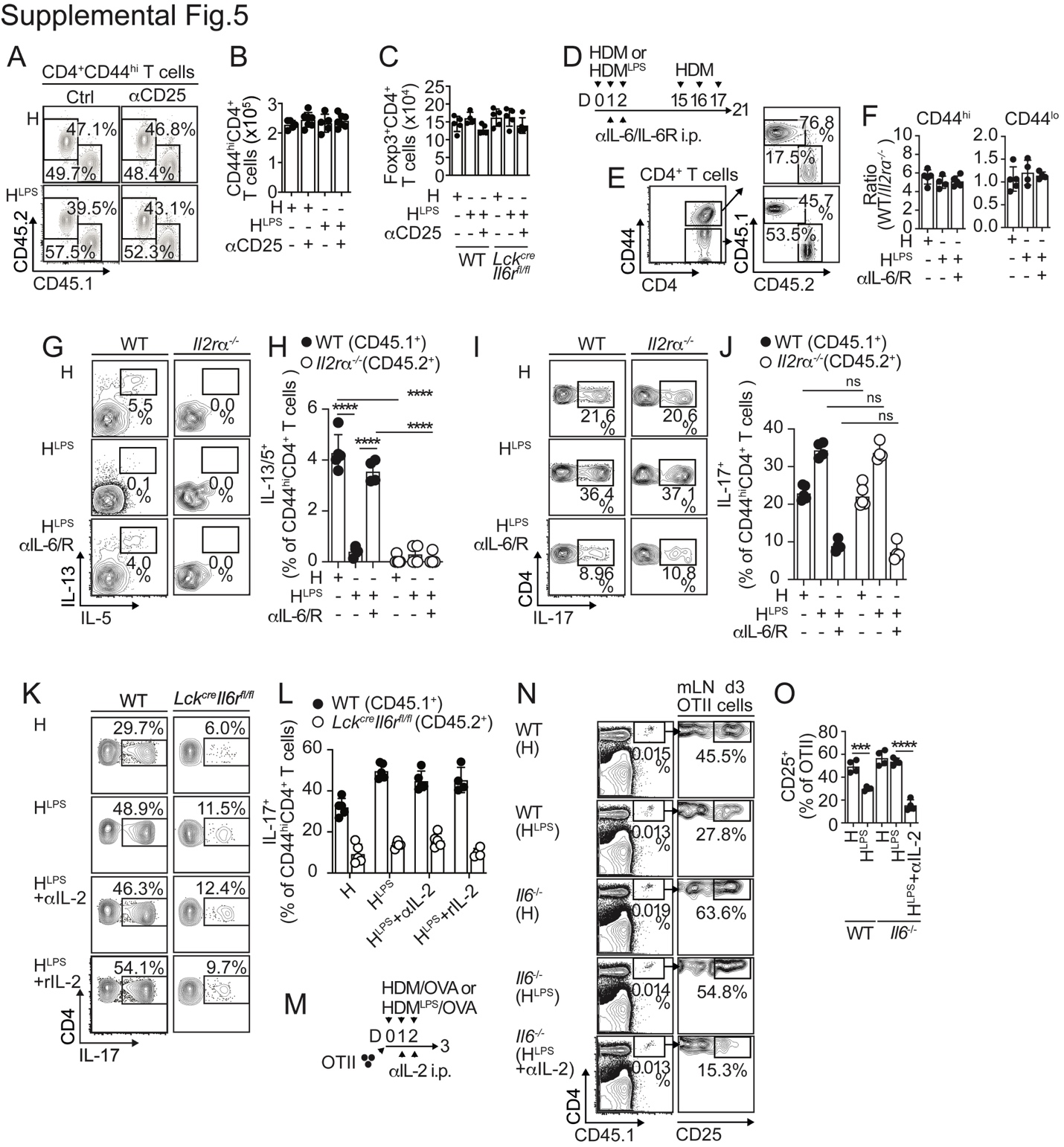


**Fig. S5. IL-2 signaling on allergen-specific T cells does not regulate polarization toward a Th17 profile.**

(**A-C**) WT:*Lck^cre^-Il6r^fl/fl^* chimeras were i.n sensitized with HDM or HDM^LPS^, i.p treated with anti-CD25 or PBS, and i.n challenged with HDM. Frequencies of CD45.1^+^ and CD45.2^+^ cells in CD44^hi^CD4^+^ T cells from the lungs (**A**). Numbers of CD44^hi^CD4^+^ T cells in the lung (**B**). Numbers of Foxp3^+^CD4^+^ T cells in the lung (**C**). (**D-J**) Irradiated B6 (CD45.1^+^) mice were reconstituted with 1:1 BM mix of B6 (CD45.1^+^) and *Il2rα^-/-^* (CD45.2^+^) donors. Chimeras were i.n sensitized with HDM or HDM^LPS^. Some mice also received 250µg anti-IL-6 and anti-IL-6R (i.p.). Mice were then challenged with HDM (**D**). Frequencies of CD45.1^+^ and CD45.2^+^ cells in CD44^hi^ and CD44^lo^ CD4^+^ T cells from the lungs (**E**). Ratio of WT to *Il2rα^-/-^* CD44^hi^ and CD44^lo^ CD4^+^ T cells (**F**). Frequencies of IL-13^+^IL-5^+^ (**G-H**) and IL-17^+^ (**I-J**) cells within the WT and *Il2rα^-/-^* CD44^hi^CD4^+^ T cell compartments in the lungs. (**K-L**) WT:*Lck^cre^-Il6r^fl/fl^* chimeras were i.n sensitized with HDM or HDM^LPS^, i.p treated with anti-IL-2 Abs (JES6-1A12 and S4B6; 250µg each), rIL-2 (60,000U) or PBS, and i.n challenged with HDM. Frequencies of IL-17^+^ cells within the WT and *Lck^cre^-Il6r^fl/fl^* CD44^hi^CD4^+^ T cell compartments in the lungs. (**M-O**) WT and *Il6^-/-^* mice were transferred with OTII.4get cells, i.n treated with HDM or HDM^LPS^ + OVA, and i.p treated with anti-IL-2 Abs or PBS (**M**). Frequencies of total (**N**) and CD25^+^ (**N-O**) OTII cells in the mLN on day 3. Data are representative of two independent experiments (mean±S.D., n=4-5, one-way Anova).
