# Supplementary material for: IL-6 prevents Th2 cell polarization by promoting SOCS3-dependent suppression of IL-2 signaling
Source: Cell Mol Immunol. 2023 Apr 12;20(6):651–65. doi: 10.1038/s41423-023-01012-1 (PMC10229632; doi:10.1038/s41423-023-01012-1)
Supplement: Supplementary file 3 — Table S2 [file 41423_2023_1012_MOESM3_ESM.docx]

| **Regulator**  Table S2 | **Expression Log Ratio** | **Predicted Activation State** |  | **Activation z-Score** | **-log10 B-H Corrected p-Value** |
| --- | --- | --- | --- | --- | --- |
| Immunoglobulin |  | complex |  | -0.154 | 21.99 |
| IL2 |  | cytokine | Activated | 2.488 | 20.96 |
| IL4 |  | cytokine |  | 1.385 | 19.26 |
| TNF |  | cytokine |  | 0.872 | 19.16 |
| Interferon alpha |  | group |  | -1.908 | 18.95 |
| IL1B |  | cytokine |  | -1.07 | 18.06 |
| IL21 | -2.827 | cytokine |  | -1.378 | 17.84 |
| IFNG | 2.033 | cytokine |  | 0.535 | 17.33 |
| STAT1 |  | transcription regulator |  | -1.132 | 16.51 |
| IL12 (complex) |  | complex |  | 0.498 | 16.10 |
| TGFB1 |  | growth factor |  | -1.03 | 15.51 |
| STAT3 |  | transcription regulator |  | -0.989 | 15.08 |
| NFATC2 |  | transcription regulator |  | -1.019 | 14.02 |
| Ige |  | complex |  | 1.65 | 13.98 |
| IFNB1 |  | cytokine |  | -0.182 | 13.28 |
| MAPK1 |  | kinase |  | 1.422 | 13.21 |
| STAT2 |  | transcription regulator |  | -1.563 | 13.21 |
| IFNAR1 |  | transmembrane receptor |  | -1.011 | 13.21 |
| SOCS1 |  | other |  | 0.958 | 13.19 |
| TBK1 |  | kinase |  | 0.25 | 13.12 |
| IL6 |  | cytokine | Inhibited | -2.24 | 12.98 |
| DNASE2 |  | enzyme |  | 1.432 | 12.98 |
| IL12 (family) |  | group |  | -0.221 | 12.74 |
| Ifnar |  | group |  | -0.491 | 12.52 |
| CREBBP |  | transcription regulator |  | -1.342 | 11.97 |
| IRF7 | -1.544 | transcription regulator | Inhibited | -2.305 | 11.53 |
| MAVS |  | other | Inhibited | -2.072 | 11.52 |
| TREX1 |  | enzyme | Activated | 2.171 | 11.52 |
| TCF3 |  | transcription regulator |  | 0.311 | 10.91 |
| STING1 |  | other |  | -0.822 | 10.83 |
| IL7 |  | cytokine |  | 1.254 | 10.78 |
| TLR9 |  | transmembrane receptor |  | -0.087 | 10.74 |
| IFNA2 |  | cytokine |  | -1.834 | 10.72 |
| EP300 |  | transcription regulator |  | -0.64 | 10.64 |
| DUSP1 |  | phosphatase |  | 0.305 | 10.62 |
| Tcf7 |  | transcription regulator |  | 0.164 | 10.55 |
| ZBTB10 |  | transcription regulator |  | 0.453 | 10.36 |
| Irgm1 |  | other |  | 1.261 | 10.30 |
| PRDM1 |  | transcription regulator |  | 0.071 | 10.20 |
| CD3 |  | complex |  | 0.888 | 10.14 |
| DUSP11 |  | phosphatase | Activated | 2.975 | 10.07 |
| IFN Beta |  | group |  | -1.642 | 9.81 |
| ID3 |  | transcription regulator |  | 1.117 | 9.70 |
| SMARCA5 |  | transcription regulator |  | -0.232 | 9.70 |
| ID2 |  | transcription regulator |  | 1.117 | 9.69 |
| SIRT1 |  | transcription regulator |  | 0.417 | 9.59 |
| TRIM24 |  | transcription regulator |  | 1.922 | 9.47 |
| IRF3 |  | transcription regulator | Inhibited | -2.406 | 9.37 |
| IL15 |  | cytokine |  | 0.543 | 9.19 |
| ACKR2 |  | G-protein coupled receptor | Activated | 2.111 | 9.19 |
| Ttc39aos1 |  | other | Activated | 2.833 | 9.16 |
| IRF8 | 1.182 | transcription regulator |  | -0.284 | 9.02 |
| KDM1A |  | enzyme |  |  | 8.97 |
| TICAM1 |  | other |  | -0.393 | 8.94 |
| IFIH1 |  | enzyme | Inhibited | -2.032 | 8.92 |
| STAT4 |  | transcription regulator |  | -1.145 | 8.86 |
| IRF9 |  | transcription regulator |  | -1.212 | 8.84 |
| IL27 |  | cytokine |  | -0.036 | 8.84 |
| IL18 | 2.095 | cytokine |  | 1.311 | 8.82 |
| APP |  | other |  | -0.159 | 8.78 |
| mir-142 |  | microRNA |  | -1.012 | 8.75 |
| MAPT |  | other |  | -0.391 | 8.75 |
| STAT6 |  | transcription regulator |  | 0.48 | 8.54 |
| FOXP3 |  | transcription regulator |  | -0.27 | 8.54 |
| EGR2 |  | transcription regulator |  | -0.106 | 8.33 |
| RNASEH2B |  | other | Activated | 3.219 | 8.31 |
| RUNX1 |  | transcription regulator |  | 0.684 | 8.30 |
| ETV6-RUNX1 |  | fusion gene/product |  | 1.976 | 8.03 |
| Ifn |  | group |  | -1.474 | 8.03 |
| LDB1 |  | transcription regulator |  | -1.606 | 7.99 |
| IFN alpha/beta |  | group |  | -0.274 | 7.97 |
| LMO2 |  | transcription regulator |  | -1.606 | 7.97 |
| CD28 |  | transmembrane receptor |  | 1.488 | 7.88 |
| MYD88 |  | other |  | 0.766 | 7.70 |
| IFNL1 |  | cytokine | Inhibited | -2.099 | 7.70 |
| cytokine |  | group |  | -0.114 | 7.68 |
| KLF2 |  | transcription regulator |  | -1.956 | 7.49 |
| TCR |  | complex |  | 0.61 | 7.44 |
| TLR4 |  | transmembrane receptor |  | 1.178 | 7.44 |
| SLC15A4 |  | transporter |  | -1.586 | 7.44 |
| ZBTB16 |  | transcription regulator |  | 1.398 | 7.34 |
| IL27RA |  | transmembrane receptor |  | 1.01 | 7.30 |
| IL1 |  | group |  | -1.244 | 7.26 |
| IFNA1/IFNA13 |  | cytokine |  | -1.196 | 7.26 |
| IL33 |  | cytokine |  | 0.948 | 7.26 |
| DDX58 |  | enzyme |  | -1.736 | 7.18 |
| RARA |  | ligand-dependent nuclear receptor |  | 1.325 | 7.05 |
| TET2 |  | enzyme |  | 1.474 | 7.01 |
| MIF |  | cytokine |  | 1.665 | 6.98 |
| IL12A |  | cytokine |  | 0.409 | 6.98 |
| SENP3 |  | peptidase |  | -1.706 | 6.98 |
| RELA |  | transcription regulator |  | 0.52 | 6.97 |
| NFkB (complex) |  | complex |  | 1.507 | 6.92 |
| BCR (complex) |  | complex |  | 0.728 | 6.85 |
| NFKB1 |  | transcription regulator | Activated | 2.245 | 6.85 |
| PARP9 |  | enzyme |  | -1.937 | 6.81 |
| IKZF1 |  | transcription regulator |  | -0.274 | 6.77 |
| EGR3 |  | transcription regulator |  | -1.284 | 6.75 |
| BACH2 |  | transcription regulator |  | -0.566 | 6.70 |
| PNPT1 |  | enzyme | Activated | 2.03 | 6.68 |
| PRL |  | cytokine |  | -1.807 | 6.67 |
| IRGM |  | enzyme | Activated | 2.132 | 6.60 |
| TBX21 | 2.475 | transcription regulator | Activated | 2.013 | 6.52 |
| SAMSN1 |  | other |  | -0.905 | 6.50 |
| CD40 |  | transmembrane receptor | Activated | 2.392 | 6.49 |
| CITED2 |  | transcription regulator |  | 1.424 | 6.47 |
| TGM2 |  | enzyme |  | -1.807 | 6.37 |
| NFATC1 |  | transcription regulator |  | 0.044 | 6.37 |
| RC3H1 |  | enzyme |  | 1.567 | 6.31 |
| BTK |  | kinase |  | -1.164 | 6.29 |
| G protein alphai |  | group |  | -1.394 | 6.27 |
| mir-24 |  | microRNA |  | 0.68 | 6.27 |
| BTNL2 |  | transmembrane receptor |  | 0.632 | 6.27 |
| IgG |  | complex |  | 1.177 | 6.27 |
| CGAS |  | enzyme | Inhibited | -2.795 | 6.26 |
| TLR3 |  | transmembrane receptor |  | -0.926 | 6.26 |
| KRAS |  | enzyme |  | 0.487 | 6.26 |
| SPI1 |  | transcription regulator |  | -1.397 | 6.25 |
| SOCS3 | -2.348 | phosphatase |  | 0.853 | 6.21 |
| ELAVL1 |  | other |  | -0.846 | 6.18 |
| STAG2 |  | other | Activated | 2.825 | 6.11 |
| EIF2AK2 |  | kinase | Inhibited | -2.238 | 6.04 |
| IL17A |  | cytokine |  | -1.297 | 6.03 |
| CSF1 |  | cytokine |  | -0.581 | 5.97 |
| MEF2A |  | transcription regulator | Inhibited | -3.156 | 5.94 |
| SOD1 |  | enzyme |  |  | 5.93 |
| PTGER4 |  | G-protein coupled receptor |  | 1.132 | 5.93 |
| TPH1 |  | enzyme | Inhibited | -2.003 | 5.90 |
| NOTCH1 |  | transcription regulator |  | 1.698 | 5.90 |
| BHLHE40 | 1.094 | transcription regulator | Activated | 2.559 | 5.85 |
| MAPK14 |  | kinase |  | 0.587 | 5.83 |
| CG |  | complex |  | -1.551 | 5.82 |
| FOXO1 |  | transcription regulator |  | -0.797 | 5.81 |
| STAT5B |  | transcription regulator |  | 0.419 | 5.81 |
| CREB1 |  | transcription regulator |  | -0.254 | 5.66 |
| CD38 |  | enzyme |  | -0.44 | 5.65 |
| SNCA |  | enzyme |  | 0.093 | 5.64 |
| IL23 |  | complex |  | -0.319 | 5.63 |
| SATB1 | -1.058 | transcription regulator | Activated | 2.634 | 5.63 |
| NR3C1 |  | ligand-dependent nuclear receptor |  | 0.324 | 5.63 |
| USP8 |  | peptidase | Activated | 2.27 | 5.63 |
| PGR |  | ligand-dependent nuclear receptor |  | 0.255 | 5.54 |
| DOCK8 |  | other |  | -0.333 | 5.52 |
| CTLA4 |  | transmembrane receptor |  | -0.843 | 5.52 |
| SASH1 |  | other |  | -0.333 | 5.47 |
| BCL6 | -1.163 | transcription regulator |  | -1.655 | 5.42 |
| TNFSF13B |  | cytokine |  | 0 | 5.35 |
| NFKB2 |  | transcription regulator |  | -0.24 | 5.35 |
| REL |  | transcription regulator |  | 0.31 | 5.32 |
| TGFBR2 |  | kinase |  | 0.153 | 5.31 |
| GFI1 |  | transcription regulator |  | 1.99 | 5.27 |
| CLEC12A |  | other |  | -1.98 | 5.20 |
| TAZ |  | enzyme |  | 0.226 | 5.19 |
| STAT5A |  | transcription regulator |  | 1.778 | 5.17 |
| Jnk |  | group |  | 0.232 | 5.15 |
| MTOR |  | kinase |  | 1.49 | 5.15 |
| IL5 |  | cytokine |  | -0.389 | 5.13 |
| GLI1 |  | transcription regulator |  | 1.487 | 5.09 |
| SMAD3 |  | transcription regulator | Inhibited | -2.05 | 5.05 |
| CD274 |  | enzyme |  | -1.872 | 5.02 |
| RNASEH2A |  | enzyme |  |  | 5.02 |
| NONO |  | transcription regulator | Inhibited | -2.105 | 5.02 |
| PIK3CG |  | kinase |  | -1.014 | 5.00 |
| RNY3 |  | other | Inhibited | -2.449 | 4.90 |
| TERT |  | enzyme | Inhibited | -2.563 | 4.88 |
| TRIM14 |  | other |  | 0.308 | 4.87 |
| TNFSF10 |  | cytokine |  | 0.235 | 4.85 |
| OSMR |  | transmembrane receptor |  |  | 4.84 |
| LYN |  | kinase |  | 0.301 | 4.82 |
| IL10 |  | cytokine |  | -1.447 | 4.81 |
| ITK |  | kinase |  | -0.289 | 4.78 |
| ERK1/2 |  | group |  | 0.666 | 4.77 |
| MAPKAP1 |  | other |  | -1.342 | 4.77 |
| IRF5 |  | transcription regulator | Inhibited | -2.921 | 4.74 |
| CD276 |  | other |  | 1.164 | 4.74 |
| CBFB |  | transcription regulator |  | -0.378 | 4.71 |
| FOXO4 |  | transcription regulator |  | -0.366 | 4.71 |
| FOXO3 |  | transcription regulator |  | -1.47 | 4.68 |
| RPTOR |  | other |  | 0.045 | 4.66 |
| PPP2CA |  | phosphatase |  | -0.114 | 4.66 |
| TNFSF11 |  | cytokine |  | 0.222 | 4.63 |
| ETV6-NTRK3 |  | fusion gene/product | Inhibited | -2 | 4.62 |
| ULBP1 |  | transmembrane receptor | Activated | 2 | 4.62 |
| CSF2 |  | cytokine |  | 0.988 | 4.58 |
| POLG2 |  | enzyme |  |  | 4.52 |
| Ap1 |  | complex |  | 0.475 | 4.50 |
| ICOS | -1.046 | transmembrane receptor |  | -0.39 | 4.49 |
| TLR7 |  | transmembrane receptor |  | -0.53 | 4.48 |
| IL1A |  | cytokine |  | -0.072 | 4.47 |
| SMARCA4 |  | transcription regulator |  | -0.176 | 4.44 |
| IFNA4 |  | cytokine |  | -0.568 | 4.44 |
| IFN type 1 |  | group |  | -0.754 | 4.44 |
| P38 MAPK |  | group |  | 0.464 | 4.39 |
| CSF3 |  | cytokine |  | -0.43 | 4.38 |
| IRF4 |  | transcription regulator |  | 0.529 | 4.38 |
| JAK1 |  | kinase |  | -1.471 | 4.37 |
| IPMK |  | kinase |  | -0.032 | 4.33 |
| PTPN11 |  | phosphatase |  | -0.005 | 4.31 |
| PTEN |  | phosphatase |  | 1.612 | 4.30 |
| TSC22D3 |  | transcription regulator |  | -0.886 | 4.28 |
| Tgf beta |  | group |  | -0.384 | 4.24 |
| NGLY1 |  | enzyme | Activated | 2.386 | 4.22 |
| IKZF2 |  | transcription regulator |  | 1.3 | 4.15 |
| ARNT |  | transcription regulator |  | 0.555 | 4.15 |
| IRF1 |  | transcription regulator |  | -0.888 | 4.15 |
| Vegf |  | group |  | -0.693 | 4.14 |
| MYC |  | transcription regulator | Activated | 2.935 | 4.14 |
| IL10RA |  | transmembrane receptor |  | 0.742 | 4.14 |
| KLF6 |  | transcription regulator |  | 0.322 | 4.14 |
| CD40LG |  | cytokine |  | 1.919 | 4.10 |
| EBI3 | 1.028 | cytokine |  | 0.734 | 4.10 |
| CD14 |  | transmembrane receptor |  | 0.218 | 4.10 |
| PARP1 |  | enzyme | Activated | 2.594 | 4.05 |
| NSD2 |  | enzyme |  | -1.342 | 4.04 |
| Histone h3 |  | group |  |  | 4.04 |
| TCF12 |  | transcription regulator |  | 0.356 | 4.03 |
| ESR2 |  | ligand-dependent nuclear receptor | Activated | 2.224 | 4.02 |
| IL23A |  | cytokine |  |  | 3.99 |
| SOX4 | 1.006 | transcription regulator |  | -0.543 | 3.98 |
| PRKAA1 |  | kinase |  | 0.24 | 3.93 |
| CAMP |  | other |  | -1.372 | 3.93 |
| CD80 |  | transmembrane receptor |  | -0.096 | 3.93 |
| KIT | -2.761 | transmembrane receptor |  |  | 3.90 |
| NFKBIA |  | transcription regulator |  | 0.506 | 3.89 |
| EGF |  | growth factor |  | -1.426 | 3.88 |
| TASL | 1.13 | enzyme |  | -0.447 | 3.86 |
| Tlr |  | group |  | 0.977 | 3.85 |
| miR-182-5p (and other miRNAs w/seed UUGGCAA) |  | mature microRNA | Activated | 2.621 | 3.85 |
| OGA |  | enzyme |  | 0.728 | 3.83 |
| IL2RG |  | transmembrane receptor |  | -0.258 | 3.81 |
| IL13 |  | cytokine |  | -0.858 | 3.80 |
| mir-96 |  | microRNA | Inhibited | -2.433 | 3.77 |
| PI3K (complex) |  | complex |  | -0.236 | 3.76 |
| IFNL4 |  | cytokine |  | -1.977 | 3.75 |
| HTT |  | transcription regulator |  |  | 3.75 |
| MAF |  | transcription regulator |  | -0.399 | 3.69 |
| IFNAR2 |  | transmembrane receptor |  |  | 3.67 |
| NFAT5 |  | transcription regulator |  | 1.127 | 3.67 |
| TP63 |  | transcription regulator |  | 0.849 | 3.66 |
| TNFSF4 |  | cytokine |  | -0.829 | 3.64 |
| ACTL6A |  | other |  |  | 3.64 |
| RAG1 |  | enzyme |  |  | 3.62 |
| mir-21 |  | microRNA |  | -0.638 | 3.61 |
| BCL11B |  | transcription regulator |  | 1.115 | 3.59 |
| IFNL3 |  | cytokine |  | -0.665 | 3.57 |
| EPOR |  | transmembrane receptor |  |  | 3.57 |
| DNMT3A |  | enzyme |  | 0.412 | 3.53 |
| SPIB |  | transcription regulator |  | -1.134 | 3.52 |
| ZBTB7B |  | transcription regulator |  | 0.849 | 3.51 |
| KLF3 |  | transcription regulator | Activated | 2.537 | 3.51 |
| F2 |  | peptidase |  | -1.093 | 3.50 |
| POMC |  | other |  | -1.099 | 3.49 |
| Akt |  | group |  | 0.352 | 3.47 |
| CTNNB1 |  | transcription regulator |  | -0.12 | 3.47 |
| IL1RN |  | cytokine | Activated | 2.155 | 3.46 |
| VCAN |  | other |  | 0.664 | 3.45 |
| TNFRSF1A |  | transmembrane receptor |  | -0.447 | 3.45 |
| IGF1 |  | growth factor |  | 0.726 | 3.45 |
| USP18 | -1.113 | peptidase |  | 1.387 | 3.45 |
| ITGB2 |  | transmembrane receptor |  | 1.664 | 3.45 |
| ATN1 |  | transcription regulator |  |  | 3.44 |
| TSC2 |  | other | Activated | 2.535 | 3.41 |
| IL12RB1 |  | transmembrane receptor |  |  | 3.40 |
| mir-181 |  | microRNA |  | -1.054 | 3.39 |
| BAK1 |  | other |  | -1.432 | 3.39 |
| IL21R |  | transmembrane receptor |  | -0.314 | 3.39 |
| S100A9 |  | other |  | 0.508 | 3.38 |
| RICTOR |  | other |  | -0.736 | 3.35 |
| EZH2 |  | transcription regulator |  | 0.228 | 3.34 |
| PRKAA2 |  | kinase |  | 1.042 | 3.33 |
| CD44 |  | other |  | 0.251 | 3.33 |
| ANXA1 |  | enzyme |  | 0.186 | 3.33 |
| FZD9 |  | G-protein coupled receptor |  | -1.131 | 3.33 |
| TNK1 |  | kinase |  | -1 | 3.33 |
| mir-183 |  | microRNA | Inhibited | -2.449 | 3.33 |
| PPP2R5C |  | other |  | -1 | 3.33 |
| NFKBIB |  | transcription regulator |  |  | 3.33 |
| FAS |  | transmembrane receptor |  | 0.555 | 3.33 |
| CXCL8 |  | cytokine |  | 0.851 | 3.33 |
| HAVCR1 |  | other |  | -0.555 | 3.33 |
| CX3CL1 |  | cytokine |  | -1.242 | 3.30 |
| ATG7 |  | enzyme |  | 0.194 | 3.30 |
| RHOA |  | enzyme |  | 0.311 | 3.30 |
| SP110 |  | transcription regulator |  | 1 | 3.30 |
| FCGR2A | -1.103 | transmembrane receptor |  | -0.557 | 3.29 |
| Secretase gamma |  | complex |  | 0.247 | 3.28 |
| mir-155 |  | microRNA | Activated | 2.25 | 3.25 |
| CD70 |  | cytokine |  |  | 3.24 |
| FABP5 |  | transporter |  | -0.339 | 3.24 |
| TP53 |  | transcription regulator |  | -0.681 | 3.23 |
| CD5 |  | transmembrane receptor |  | -0.97 | 3.21 |
| JUND |  | transcription regulator |  | -0.221 | 3.18 |
| RGS10 |  | enzyme |  |  | 3.18 |
| PML-RARA |  | fusion gene/product |  |  | 3.17 |
| Klrk1 |  | transmembrane receptor |  | 1.257 | 3.17 |
| IL3 |  | cytokine |  | 1.136 | 3.17 |
| IFNA14 |  | cytokine |  | -0.882 | 3.16 |
| IKBKB |  | kinase |  | 0.252 | 3.15 |
| POU2AF1 | -1.257 | transcription regulator |  | -1.944 | 3.13 |
| CD4 |  | transmembrane receptor |  | 0.349 | 3.13 |
| IL12B |  | cytokine |  | 1.512 | 3.11 |
| GIP |  | other |  | -1.664 | 3.11 |
| FOXC1 |  | transcription regulator |  | -1.238 | 3.10 |
| PIM2 |  | kinase |  | 1.131 | 3.08 |
| MGA |  | transcription regulator |  | 1.633 | 3.08 |
| TNFRSF8 | 1.015 | transmembrane receptor |  | -1.633 | 3.08 |
| SPP1 |  | cytokine |  | -0.304 | 3.06 |
| NOS2 |  | enzyme |  | -1.287 | 3.06 |
| immune complex |  | complex |  |  | 3.04 |
| Duxbl1 |  | other |  |  | 3.04 |
| SPIC |  | transcription regulator |  |  | 3.04 |
| GAPDH |  | enzyme |  | 0.447 | 3.04 |
| TCF4 | 1.51 | transcription regulator |  | -1.667 | 3.02 |
| MAP3K8 |  | kinase |  | 0.757 | 3.01 |
| HNRNPU |  | transporter |  | 1.664 | 3.01 |
| MVP |  | other |  |  | 3.01 |
| AGT |  | growth factor |  | -0.76 | 3.00 |
| JUN | -1.163 | transcription regulator |  | 1.849 | 3.00 |
| TNFSF15 |  | cytokine | Activated | 2.188 | 2.99 |
| LDL |  | complex |  | 0.45 | 2.98 |
| HGF |  | growth factor | Inhibited | -2.458 | 2.95 |
| TLR2 |  | transmembrane receptor |  | 1.645 | 2.95 |
| KEAP1 |  | transcription regulator |  | 1.253 | 2.94 |
| CREM |  | transcription regulator |  | -0.633 | 2.94 |
| HIF1A |  | transcription regulator |  | -0.039 | 2.94 |
| BMP10 |  | growth factor |  | 1.25 | 2.93 |
| PDLIM2 |  | other |  | 1.134 | 2.93 |
| HMGB1 |  | transcription regulator |  | 0.422 | 2.93 |
| OSM |  | cytokine |  | -0.184 | 2.92 |
| Tnf (family) |  | group |  | 0.046 | 2.92 |
| CEBPB |  | transcription regulator | Inhibited | -2.764 | 2.92 |
| CD3E |  | transmembrane receptor |  |  | 2.92 |
| STAT5a/b |  | group |  | 0.876 | 2.91 |
| GSE1 |  | other |  |  | 2.91 |
| DUSP16 |  | phosphatase |  |  | 2.91 |
| CYLD |  | transcription regulator |  | -0.52 | 2.91 |
| ZBED2 |  | transcription regulator |  |  | 2.91 |
| FCGR3A/FCGR3B |  | transmembrane receptor |  |  | 2.91 |
| IL12RB2 |  | transmembrane receptor |  |  | 2.91 |
| EGLN1 |  | enzyme |  | 1.633 | 2.90 |
| DYSF |  | other |  |  | 2.89 |
| NADPH oxidase |  | complex |  |  | 2.89 |
| IFNK |  | cytokine |  | 0.943 | 2.89 |
| SUPT20H |  | other |  | 1 | 2.89 |
| STAT4 dimer |  | complex |  |  | 2.88 |
| ANO6 |  | ion channel |  |  | 2.88 |
| IFIT1B | -1.581 | other |  |  | 2.88 |
| Raet1a |  | other |  |  | 2.88 |
| GAB3 |  | other |  |  | 2.88 |
| RNA polymerase II |  | complex |  |  | 2.88 |
| JAK |  | group | Inhibited | -2 | 2.88 |
| ARHGAP21 |  | other |  | 1.342 | 2.88 |
| NFATC3 |  | transcription regulator |  |  | 2.88 |
| IL4R |  | transmembrane receptor |  | 1.039 | 2.88 |
| IL6ST |  | transmembrane receptor | Inhibited | -2.2 | 2.88 |
| NCOA2 |  | transcription regulator |  | -0.547 | 2.84 |
| IGHM |  | transmembrane receptor |  |  | 2.84 |
| IL36B |  | cytokine |  |  | 2.83 |
| CD81 |  | other |  |  | 2.83 |
| NCOR1 |  | transcription regulator | Activated | 2.621 | 2.83 |
| TOX |  | transcription regulator |  | 1 | 2.83 |
| TSLP |  | cytokine |  | 1.452 | 2.82 |
| CXCL12 |  | cytokine |  | -0.736 | 2.81 |
| FLT3 |  | kinase |  |  | 2.80 |
| ERK |  | group |  | 0.562 | 2.79 |
| Stat1-Stat2 |  | complex |  |  | 2.79 |
| RC3H2 |  | enzyme |  |  | 2.79 |
| NR4A1 |  | ligand-dependent nuclear receptor |  | 1.077 | 2.77 |
| TRA |  | transmembrane receptor |  |  | 2.77 |
| PRKACA |  | kinase |  | 0 | 2.76 |
| IFNLR1 |  | transmembrane receptor |  |  | 2.76 |
| EPAS1 |  | transcription regulator |  | -0.426 | 2.73 |
| ISG15 |  | other | Activated | 2.207 | 2.72 |
| ZC3H12A |  | enzyme |  | -0.867 | 2.71 |
| HRAS |  | enzyme |  | -1.757 | 2.71 |
| FLT3LG |  | cytokine | Activated | 2.421 | 2.71 |
| CHD4 |  | enzyme | Activated | 2.236 | 2.71 |
| Map3k7 |  | kinase |  | 0.168 | 2.71 |
| MAPK9 |  | kinase |  | 0.459 | 2.70 |
| MET |  | kinase |  | -0.378 | 2.70 |
| FYN |  | kinase |  | 0.555 | 2.69 |
| ADAM10 |  | peptidase | Inhibited | -2 | 2.68 |
| IFN Lambda |  | group |  |  | 2.68 |
| TXK |  | kinase |  |  | 2.68 |
| MAPK3 |  | kinase |  | -0.928 | 2.68 |
| APOH |  | transporter |  |  | 2.68 |
| IKBKG |  | kinase |  | -0.989 | 2.67 |
| ECSIT |  | transcription regulator |  | -1.109 | 2.66 |
| TXN |  | enzyme | Inhibited | -2 | 2.66 |
| CALCA |  | other |  | -1.633 | 2.66 |
| Srgn |  | other |  | 0.447 | 2.66 |
| CISH | 1.697 | other |  | -1.342 | 2.66 |
| EPO |  | cytokine |  | -1.367 | 2.66 |
| Mapk |  | group |  | 0.218 | 2.64 |
| LEP |  | growth factor |  | -0.177 | 2.64 |
| Pka |  | complex |  | 0 | 2.63 |
| NKX2-3 |  | transcription regulator |  | 0.681 | 2.63 |
| NEIL2 |  | enzyme |  |  | 2.63 |
| AHR | 1.072 | ligand-dependent nuclear receptor |  | -0.623 | 2.63 |
| PPARGC1A |  | transcription regulator |  | 0.397 | 2.63 |
| JAK3 |  | kinase |  | 1.342 | 2.63 |
| WNT3A |  | cytokine |  | -0.488 | 2.62 |
| S100A8 |  | other |  | 0.707 | 2.62 |
| GATA4 |  | transcription regulator |  | 0.673 | 2.62 |
| LIF |  | cytokine |  | -0.72 | 2.61 |
| ERBB2 |  | kinase |  | 0.163 | 2.61 |
| LRP5 |  | transmembrane receptor |  | 0 | 2.59 |
| STAT1/3/5 dimer |  | complex |  |  | 2.59 |
| CCL5 |  | cytokine |  | -0.447 | 2.59 |
| TCIRG1 |  | enzyme |  |  | 2.59 |
| PRKCE |  | kinase |  | 0.128 | 2.59 |
| HLA-G |  | other |  |  | 2.59 |
| HMG20A |  | transcription regulator |  | -0.378 | 2.58 |
| CCL2 |  | cytokine |  | 0.218 | 2.56 |
| RAC2 |  | enzyme |  |  | 2.56 |
| PRKDC |  | kinase |  |  | 2.56 |
| IRAK4 |  | kinase |  | 1.078 | 2.56 |
| MAP3K7 |  | kinase |  | -0.218 | 2.56 |
| BATF |  | transcription regulator |  |  | 2.56 |
| SQSTM1 |  | transcription regulator |  | -1 | 2.56 |
| SPHK1 |  | kinase |  | -0.803 | 2.53 |
| CD86 | 2.676 | transmembrane receptor |  | 0.494 | 2.53 |
| PPARG |  | ligand-dependent nuclear receptor | Inhibited | -2.597 | 2.51 |
| CD69 |  | transmembrane receptor |  | 1 | 2.51 |
| PSMD14 |  | peptidase |  |  | 2.50 |
| IgG2a |  | complex |  |  | 2.49 |
| PIM3 |  | kinase |  |  | 2.49 |
| TIGIT | -2.81 | other |  |  | 2.49 |
| BAX |  | transporter |  | -1.432 | 2.49 |
| PF4 |  | cytokine |  | 0.603 | 2.49 |
| Focal adhesion kinase |  | group |  | 1.964 | 2.47 |
| TGFB2 |  | growth factor |  | -1.026 | 2.47 |
| TG | 1.125 | other |  |  | 2.47 |
| WAS |  | other |  |  | 2.47 |
| BRD4 |  | kinase | Activated | 2.2 | 2.44 |
| RUNX1-RUNX1T1 |  | fusion gene/product |  |  | 2.42 |
| HSPD1 |  | enzyme |  | -0.246 | 2.42 |
| ZC3H12C |  | other |  | -0.152 | 2.42 |
| TRB |  | transmembrane receptor |  |  | 2.42 |
| THRB |  | ligand-dependent nuclear receptor |  | -0.277 | 2.42 |
| miR-30c-5p (and other miRNAs w/seed GUAAACA) |  | mature microRNA |  | 1.181 | 2.41 |
| AIRE |  | transcription regulator |  |  | 2.41 |
| SCAVENGER receptor CLASS A |  | group |  |  | 2.41 |
| LTA | 1.052 | cytokine |  | 1.474 | 2.40 |
| FOS |  | transcription regulator | Activated | 2.415 | 2.40 |
| AMPK |  | complex |  | -0.342 | 2.39 |
| HFE |  | transmembrane receptor |  | -0.452 | 2.39 |
| TREM1 |  | transmembrane receptor |  | 1.154 | 2.38 |
| HAVCR2 |  | other |  | -1.103 | 2.38 |
| CNOT7 |  | transcription regulator |  |  | 2.38 |
| PIK3R1 |  | kinase |  | 0.128 | 2.37 |
| FADD |  | other |  | -1.673 | 2.37 |
| HNF1B |  | transcription regulator |  | 1.387 | 2.35 |
| TFRC |  | transporter |  | 1.673 | 2.35 |
| IFNL2 |  | other |  |  | 2.33 |
| TAP1 |  | transporter |  |  | 2.33 |
| BDNF |  | growth factor |  | -0.803 | 2.33 |
| PRKCA |  | kinase |  | -1.117 | 2.32 |
| ROCK2 |  | kinase |  |  | 2.32 |
| Insulin |  | group | Inhibited | -2.024 | 2.31 |
| UCP1 |  | transporter |  | 1.463 | 2.31 |
| PRKCZ |  | kinase |  | 0.152 | 2.29 |
| MARK2 |  | kinase |  | 0 | 2.29 |
| IKBKE |  | kinase |  | -0.655 | 2.29 |
| PML |  | transcription regulator |  | -1.348 | 2.27 |
| Hdac |  | group |  | 1.722 | 2.26 |
| TYROBP |  | transmembrane receptor |  | 1.982 | 2.26 |
| Stat3-Stat3 |  | complex |  | -1.177 | 2.26 |
| TNFSF14 |  | cytokine |  | 0.851 | 2.26 |
| DLL1 |  | enzyme |  | 1.103 | 2.26 |
| CDC42 |  | enzyme |  |  | 2.26 |
| MUC13 |  | other |  |  | 2.26 |
| NLRP12 |  | other |  | -0.283 | 2.26 |
| AIM2 |  | other |  | 1.98 | 2.26 |
| Il12 receptor |  | complex |  |  | 2.25 |
| NLRP6 |  | G-protein coupled receptor |  |  | 2.25 |
| RAB11FIP3 |  | other |  |  | 2.25 |
| TPP2 |  | peptidase |  |  | 2.25 |
| PSEN1 |  | peptidase |  | -0.063 | 2.25 |
| RARG | -1.375 | ligand-dependent nuclear receptor |  | 0.277 | 2.25 |
| ATF2 |  | transcription regulator |  |  | 2.25 |
| FGF1 |  | growth factor |  | -0.705 | 2.24 |
| NR4A2 |  | ligand-dependent nuclear receptor |  | -0.487 | 2.23 |
| RNASEL |  | enzyme |  |  | 2.23 |
| PRKCQ |  | kinase |  | 1.929 | 2.23 |
| MSC |  | transcription regulator |  | -1.342 | 2.22 |
| TAF4 |  | transcription regulator |  |  | 2.21 |
| Integrin |  | complex |  |  | 2.20 |
| Foxo |  | group |  |  | 2.20 |
| USP1 |  | peptidase |  |  | 2.20 |
| TNFRSF6B |  | transmembrane receptor |  |  | 2.20 |
| RAS |  | group |  | -0.208 | 2.20 |
| NLRP3 |  | other |  | 0.543 | 2.20 |
| POU2F2 |  | transcription regulator |  | -1.633 | 2.20 |
| CD200R1 |  | transmembrane receptor |  | 1.342 | 2.20 |
| CNTF |  | cytokine |  | -1.77 | 2.19 |
| CHUK |  | kinase |  | 0.386 | 2.19 |
| CASZ1 |  | enzyme |  | 1.091 | 2.19 |
| SMARCB1 |  | transcription regulator |  | -0.447 | 2.16 |
| NMDA Receptor |  | complex |  |  | 2.14 |
| XRCC6 |  | enzyme |  |  | 2.14 |
| TRAF5 |  | transporter |  |  | 2.14 |
| RARB |  | ligand-dependent nuclear receptor |  | 0.478 | 2.14 |
| IRF2 |  | transcription regulator |  | 1.414 | 2.14 |
| CDKN2A |  | transcription regulator |  | -0.421 | 2.13 |
| Pdgf (complex) |  | complex |  | -1.202 | 2.12 |
| F2RL1 | -1.839 | G-protein coupled receptor |  |  | 2.12 |
| PRKCB |  | kinase |  | 0.447 | 2.12 |
| ATF3 |  | transcription regulator |  | 0.061 | 2.12 |
| C10orf99 |  | cytokine |  | 0 | 2.12 |
| PRKCI |  | kinase |  | -0.218 | 2.08 |
| Dexamethasone-GR |  | complex |  |  | 2.08 |
| p38 Sapk |  | group |  |  | 2.08 |
| CYP |  | group |  |  | 2.08 |
| Mucin |  | group |  |  | 2.08 |
| ESR1 | 1.451 | ligand-dependent nuclear receptor |  | -0.66 | 2.08 |
| EGOT |  | other |  |  | 2.08 |
| PAEP |  | other |  |  | 2.08 |
| ICAM5 |  | other |  |  | 2.08 |
| LAMP1 |  | other |  |  | 2.08 |
| IL31RA |  | transmembrane receptor |  |  | 2.08 |
| HLA-B |  | transmembrane receptor |  |  | 2.08 |
| MAPK8 |  | kinase |  | 0.7 | 2.08 |
| USF2 |  | transcription regulator |  | -0.254 | 2.08 |
| ADRB |  | group |  | 1.89 | 2.07 |
| ETS1 |  | transcription regulator |  | 0.277 | 2.06 |
| AGTR1 |  | G-protein coupled receptor |  |  | 2.06 |
| CIITA |  | transcription regulator |  | -1.086 | 2.06 |
| ATF1 |  | transcription regulator |  |  | 2.06 |
| RHO |  | G-protein coupled receptor |  | 0.447 | 2.06 |
| JAK1/2 |  | group |  | 0.447 | 2.06 |
| IKZF3 |  | transcription regulator | Activated | 2.219 | 2.06 |
| RAG2 |  | enzyme |  |  | 2.04 |
| TNFRSF1B |  | transmembrane receptor |  | -1.342 | 2.04 |
| IFNW1 |  | cytokine |  |  | 2.03 |
| ORAI1 |  | ion channel |  |  | 2.03 |
| ZBP1 |  | other |  |  | 2.03 |
| KLRC4-KLRK1/KLRK1 |  | transmembrane receptor |  |  | 2.03 |
| Klra7 (includes others) |  | transmembrane receptor |  |  | 2.03 |
| IFNE |  | cytokine |  | 1.091 | 2.03 |
| CD2 |  | transmembrane receptor |  |  | 2.03 |
| CYP19A1 |  | enzyme |  | -1.616 | 2.02 |
| LCK |  | kinase |  | 1.461 | 2.02 |
| PRKD1 |  | kinase |  | 1.387 | 2.02 |
| TARDBP |  | transcription regulator |  | -0.152 | 2.02 |
| PAX7 |  | transcription regulator |  |  | 2.02 |
| EPHA2 |  | kinase |  | -1.091 | 2.00 |
| mir-223 |  | microRNA |  | -1.134 | 2.00 |
| HNRNPA2B1 |  | other |  |  | 2.00 |
| PTPRC |  | phosphatase |  |  | 2.00 |
| CRH |  | cytokine |  | -1.066 | 2.00 |
| JAG1 |  | growth factor |  | 0.254 | 2.00 |
| PDCD1 |  | transmembrane receptor |  | -1.998 | 2.00 |
| SREBF1 |  | transcription regulator |  | -0.653 | 1.99 |
| Stat5 dimer |  | complex |  |  | 1.98 |
| PER1 |  | transcription regulator |  |  | 1.98 |
| Histone h4 |  | group |  |  | 1.95 |
| PIEZO1 |  | ion channel |  |  | 1.95 |
| SH2B3 |  | other |  |  | 1.95 |
| ZNF395 |  | transcription regulator |  |  | 1.95 |
| AATF |  | transcription regulator |  |  | 1.95 |
| BTG1 |  | transcription regulator |  |  | 1.95 |
| Hbb-b2 |  | other |  |  | 1.94 |
| DPP4 |  | peptidase |  | -0.686 | 1.94 |
| TNFSF9 |  | cytokine |  |  | 1.94 |
| PLCG2 |  | enzyme |  | 0.492 | 1.94 |
| MAP3K5 |  | kinase |  |  | 1.94 |
| MAP2K6 |  | kinase |  |  | 1.94 |
| MAPK8IP1 |  | other |  |  | 1.94 |
| GNAQ |  | enzyme |  | -0.698 | 1.92 |
| ZFTA-RELA |  | fusion gene/product | Inhibited | -2 | 1.92 |
| EOMES | 1.788 | transcription regulator |  | -0.6 | 1.91 |
| Creb |  | group |  | -0.555 | 1.91 |
| MAP2K1/2 |  | group |  | -0.555 | 1.90 |
| RORC | -2.169 | ligand-dependent nuclear receptor |  | -0.93 | 1.90 |
| EHMT1 |  | transcription regulator |  | 0 | 1.90 |
| JUNB |  | transcription regulator |  | 0.099 | 1.90 |
| SCGB1A1 |  | cytokine |  |  | 1.89 |
| Tnfsf9 |  | other |  |  | 1.89 |
| IL2RA | 1.576 | transmembrane receptor |  |  | 1.89 |
| OLR1 |  | transmembrane receptor |  | -0.145 | 1.89 |
| INSR |  | kinase |  | -0.239 | 1.87 |
| AR |  | ligand-dependent nuclear receptor |  | -0.567 | 1.87 |
| CAV1 |  | transmembrane receptor |  | -0.412 | 1.87 |
| GATA1 |  | transcription regulator |  | -0.196 | 1.87 |
| IL24 |  | cytokine |  | 1.96 | 1.86 |
| SMAD2 |  | transcription regulator |  | -1.751 | 1.85 |
| TRIM21 |  | enzyme |  |  | 1.84 |
| BMPER |  | other |  |  | 1.84 |
| LGALS9 |  | other |  |  | 1.84 |
| SOCS2 |  | other |  |  | 1.84 |
| FASLG | 1.271 | cytokine |  | -1 | 1.84 |
| CCL18 |  | cytokine |  |  | 1.84 |
| RAC1 |  | enzyme |  | 0.246 | 1.84 |
| ENPP1 |  | enzyme |  |  | 1.84 |
| SH3RF1 |  | enzyme |  |  | 1.84 |
| PDE4D |  | enzyme |  |  | 1.84 |
| Calcineurin B |  | group |  |  | 1.84 |
| MAP3K14 |  | kinase |  | 0.537 | 1.84 |
| miR-494-3p (miRNAs w/seed GAAACAU) |  | mature microRNA |  |  | 1.84 |
| NLRX1 |  | other |  | 0.728 | 1.84 |
| LIMIT |  | other |  |  | 1.84 |
| SERPINA4 |  | other |  |  | 1.84 |
| CASP4 |  | peptidase |  |  | 1.84 |
| CTSE |  | peptidase |  |  | 1.84 |
| POU5F1 |  | transcription regulator |  | 0.486 | 1.84 |
| ULBP2 |  | transmembrane receptor |  |  | 1.84 |
| STRA6 | 2.099 | transporter |  |  | 1.84 |
| GJA8 |  | transporter |  |  | 1.84 |
| TGFB3 | -2.259 | growth factor |  | -1.293 | 1.83 |
| ATF4 |  | transcription regulator |  | -0.33 | 1.83 |
| Hsp70 |  | group |  |  | 1.82 |
| H2AB3 (includes others) |  | other |  | 0 | 1.82 |
| POM121/POM121C |  | other |  |  | 1.81 |
| TNFRSF10A |  | transmembrane receptor |  |  | 1.81 |
| EDN1 |  | cytokine |  | -0.051 | 1.81 |
| PSMB11 |  | peptidase |  | 0.816 | 1.79 |
| POU2F1 |  | transcription regulator |  |  | 1.79 |
| PARP2 |  | enzyme |  |  | 1.77 |
| MAP3K3 |  | kinase |  |  | 1.77 |
| BRD2 |  | kinase |  |  | 1.77 |
| mir-154 |  | microRNA |  |  | 1.77 |
| LGALS1 |  | other |  | 0.365 | 1.77 |
| ATG5 |  | other |  | 0.447 | 1.77 |
| IL31 |  | other |  |  | 1.77 |
| TGIF1 |  | transcription regulator |  |  | 1.77 |
| TAF4B |  | transcription regulator |  |  | 1.77 |
| FHL2 |  | transcription regulator |  | -0.762 | 1.77 |
| SIGIRR |  | transmembrane receptor |  |  | 1.77 |
| CCN1 |  | other |  | 0.277 | 1.76 |
| MUC1 |  | other |  | -1.109 | 1.76 |
| BLM |  | enzyme |  |  | 1.75 |
| PCM1-JAK2 |  | fusion gene/product |  |  | 1.75 |
| ADRB2 |  | G-protein coupled receptor |  |  | 1.75 |
| VIPR2 |  | G-protein coupled receptor |  |  | 1.75 |
| ROCK |  | group |  | 1.067 | 1.75 |
| CD3 group |  | group |  | 1.985 | 1.75 |
| mir-30 |  | microRNA |  | -0.351 | 1.75 |
| MIR17HG | 1.162 | other |  | -0.254 | 1.75 |
| VTCN1 |  | other |  |  | 1.75 |
| CLTC |  | other |  |  | 1.75 |
| Gm20703 |  | other |  |  | 1.75 |
| SOX18 |  | transcription regulator |  |  | 1.75 |
| TBL1X |  | transcription regulator |  |  | 1.75 |
| IL23R |  | transmembrane receptor |  |  | 1.75 |
| NFAT (complex) |  | complex |  |  | 1.74 |
| CHI3L1 |  | enzyme |  |  | 1.74 |
| P2RY6 |  | G-protein coupled receptor |  |  | 1.74 |
| JUN/JUNB/JUND |  | group |  |  | 1.74 |
| SLC9A3 |  | ion channel |  |  | 1.74 |
| SBDS |  | other |  |  | 1.74 |
| PROCR |  | other |  |  | 1.74 |
| ICOSLG/LOC102723996 |  | other |  |  | 1.74 |
| CTSS |  | peptidase |  |  | 1.74 |
| MTA2 |  | transcription regulator |  |  | 1.74 |
| FN1 |  | enzyme |  | 1.414 | 1.74 |
| MTORC1 |  | complex |  | 1.091 | 1.73 |
| TNFAIP3 |  | enzyme |  | -1.067 | 1.73 |
| NOX4 |  | enzyme |  | 0.762 | 1.73 |
| Hsp90 |  | group |  | -0.147 | 1.73 |
| SAFB |  | other |  | 1 | 1.73 |
| DCN |  | other |  | 0.152 | 1.73 |
| NEDD9 |  | other |  | 1.342 | 1.73 |
| EIF2S1 |  | translation regulator |  |  | 1.73 |
| ICAM1 |  | transmembrane receptor |  | 1.154 | 1.73 |
| MED1 |  | transcription regulator |  |  | 1.73 |
| SOX9 |  | transcription regulator |  | -1.633 | 1.73 |
| DICER1 |  | enzyme |  | -0.846 | 1.71 |
| CAT |  | enzyme |  | 0.218 | 1.71 |
| BIRC5 |  | other |  |  | 1.71 |
| PTPRJ |  | phosphatase | Activated | 2 | 1.71 |
| CEBPE |  | transcription regulator |  |  | 1.71 |
| AIP |  | transcription regulator |  | 0.218 | 1.71 |
| CBL |  | transcription regulator |  |  | 1.71 |
| miR-203a-3p (and other miRNAs w/seed UGAAAUG) |  | mature microRNA |  |  | 1.71 |
| SFN |  | other |  |  | 1.71 |
| VDR |  | transcription regulator |  | -0.936 | 1.71 |
| RAF1 |  | kinase |  | -0.57 | 1.70 |
| NKX3-1 |  | transcription regulator |  |  | 1.69 |
| NFE2L2 |  | transcription regulator |  | -0.958 | 1.68 |
| XCL1 |  | cytokine |  |  | 1.68 |
| IRS1 |  | enzyme |  | -0.329 | 1.68 |
| BLVRA |  | enzyme |  |  | 1.68 |
| UBA7 |  | enzyme |  |  | 1.68 |
| DRD5 |  | G-protein coupled receptor |  |  | 1.68 |
| GPR183 |  | G-protein coupled receptor |  |  | 1.68 |
| HVCN1 |  | ion channel |  |  | 1.68 |
| MAP3K12 |  | kinase |  |  | 1.68 |
| PIM1 |  | kinase |  | 1.131 | 1.68 |
| mir-338 |  | microRNA |  |  | 1.68 |
| MEMO1 |  | other |  |  | 1.68 |
| SHANK3 |  | other |  |  | 1.68 |
| CTSZ |  | peptidase |  |  | 1.68 |
| HP |  | peptidase |  |  | 1.68 |
| CAPN1 |  | peptidase |  |  | 1.68 |
| PPP3CA |  | phosphatase |  |  | 1.68 |
| ARID5A |  | transcription regulator |  |  | 1.68 |
| SMARCC2 |  | transcription regulator |  |  | 1.68 |
| MNT |  | transcription regulator |  | 0 | 1.68 |
| IL22RA2 |  | transmembrane receptor |  |  | 1.68 |
| CD160 |  | transmembrane receptor |  |  | 1.68 |
| PDYN |  | transporter |  |  | 1.68 |
| Hbb-b1 |  | transporter |  | -0.218 | 1.68 |
| RASSF1 |  | other |  | 0.447 | 1.66 |
| MAPKAPK2 |  | kinase |  | 1.103 | 1.65 |
| GNAI2 |  | enzyme |  |  | 1.64 |
| USP22 |  | peptidase |  | 0.762 | 1.63 |
| Ifn gamma |  | complex |  | -0.497 | 1.61 |
| CXCR3 | 1.162 | G-protein coupled receptor |  |  | 1.61 |
| Ubiquitin |  | group |  |  | 1.61 |
| TRPV4 |  | ion channel |  |  | 1.61 |
| SPHK2 |  | kinase |  |  | 1.61 |
| mir-29 |  | microRNA |  | 0.356 | 1.61 |
| VTN |  | other |  |  | 1.61 |
| CFLAR |  | other |  |  | 1.61 |
| SLC22A5 |  | transporter |  |  | 1.61 |
| ARID1A |  | transcription regulator |  |  | 1.60 |
| CXCL13 |  | cytokine |  |  | 1.60 |
| TLR2/3/4/9 |  | group |  |  | 1.60 |
| KCNA3 |  | ion channel |  |  | 1.60 |
| mir-217 |  | microRNA |  |  | 1.60 |
| LRRC32 |  | other |  |  | 1.60 |
| PHF21A |  | other |  |  | 1.60 |
| RANBP9 |  | other |  |  | 1.60 |
| ATAD3A |  | other |  |  | 1.60 |
| IL9R |  | transmembrane receptor |  |  | 1.60 |
| SLC6A1 |  | transporter |  |  | 1.60 |
| IL7R |  | transmembrane receptor |  | -1.109 | 1.60 |
| BRCA1 |  | transcription regulator |  | -1.423 | 1.59 |
| ASPSCR1-TFE3 |  | fusion gene/product |  | -0.447 | 1.58 |
| collagenase |  | group |  |  | 1.58 |
| Fc gamma receptor |  | group |  |  | 1.58 |
| MAPK11 |  | kinase |  |  | 1.58 |
| TIRAP |  | other |  |  | 1.58 |
| ZEB1 |  | transcription regulator |  | 0.152 | 1.58 |
| MYCN |  | transcription regulator |  | 1.795 | 1.57 |
| LAMA4 |  | enzyme |  | 1.342 | 1.56 |
| TAB1 |  | enzyme |  |  | 1.55 |
| Fgf |  | group |  |  | 1.55 |
| LEF1 |  | transcription regulator |  | 1.342 | 1.55 |
| STAR |  | transporter |  | 0 | 1.55 |
| SCD |  | enzyme |  | 0.447 | 1.54 |
| Stat1 dimer |  | complex |  |  | 1.54 |
| Adaptor protein 2 |  | complex |  |  | 1.54 |
| IL25 |  | cytokine |  | 0.577 | 1.54 |
| TNFSF18 |  | cytokine |  |  | 1.54 |
| Ifnz (includes others) |  | cytokine |  |  | 1.54 |
| Fcor |  | enzyme |  |  | 1.54 |
| PDE3B |  | enzyme |  |  | 1.54 |
| ADAR |  | enzyme |  |  | 1.54 |
| ENTPD1 |  | enzyme |  |  | 1.54 |
| SLC9A1 |  | ion channel |  |  | 1.54 |
| LAMTOR1 |  | other |  |  | 1.54 |
| Traj18 |  | other |  |  | 1.54 |
| LNX2 |  | other |  |  | 1.54 |
| OSTM1 |  | other |  |  | 1.54 |
| MMP7 |  | peptidase |  |  | 1.54 |
| TRIM28 |  | transcription regulator |  |  | 1.54 |
| NEUROG1 |  | transcription regulator |  | 0 | 1.54 |
| KLF7 |  | transcription regulator |  |  | 1.54 |
| HIVEP3 |  | transcription regulator |  |  | 1.54 |
| ELF2 |  | transcription regulator |  |  | 1.54 |
| KLRD1 |  | transmembrane receptor |  |  | 1.54 |
| SLC7A5 |  | transporter |  |  | 1.54 |
| SYVN1 |  | transporter |  | 0.816 | 1.53 |
| THPO |  | cytokine |  | -0.152 | 1.53 |
| Nfat (family) |  | group |  | 1 | 1.53 |
| TYK2 |  | kinase |  |  | 1.53 |
| OSCAR |  | other |  |  | 1.53 |
| MALT1 |  | peptidase |  |  | 1.53 |
| NOTCH4 |  | transcription regulator |  |  | 1.53 |
| ZBTB7A |  | transcription regulator |  |  | 1.53 |
| DAXX |  | transcription regulator |  |  | 1.53 |
| SGK1 | -1.153 | kinase |  | -1.982 | 1.52 |
| CEBPA | -1.947 | transcription regulator |  | 1.446 | 1.52 |
| TRAF3 |  | enzyme |  | -0.2 | 1.52 |
| LIPE |  | enzyme |  |  | 1.51 |
| IKK (complex) |  | complex |  |  | 1.50 |
| Notch |  | group |  | 0.339 | 1.50 |
| mir-126 |  | microRNA |  |  | 1.50 |
| XBP1 |  | transcription regulator | Inhibited | -2.176 | 1.50 |
| PHB |  | transcription regulator |  |  | 1.50 |
| PROX1 |  | transcription regulator |  |  | 1.50 |
| EGR1 |  | transcription regulator |  | 0.629 | 1.49 |
| Growth hormone |  | group |  | -0.57 | 1.48 |
| PDGF BB |  | complex |  | -1.018 | 1.48 |
| ALOX12 |  | enzyme |  |  | 1.48 |
| DOK1 |  | kinase |  |  | 1.48 |
| SERPINA3 |  | other |  |  | 1.48 |
| FYB1 |  | other |  |  | 1.48 |
| ELN |  | other |  |  | 1.48 |
| MAP2K1 |  | kinase |  | -0.928 | 1.47 |
| TRIB3 | -1.217 | kinase |  |  | 1.47 |
| miR-221-3p (and other miRNAs w/seed GCUACAU) |  | mature microRNA |  |  | 1.47 |
| NCSTN |  | peptidase |  |  | 1.47 |
| RBCK1 |  | transcription regulator |  |  | 1.47 |
| 26s Proteasome |  | complex |  | 0.634 | 1.45 |
| GATA3 |  | transcription regulator | Inhibited | -2.406 | 1.45 |
| IL20 |  | cytokine |  |  | 1.45 |
| RNASE2 |  | enzyme |  |  | 1.45 |
| GNB2 |  | enzyme |  |  | 1.45 |
| AKT1 |  | kinase |  | 0.966 | 1.45 |
| VIP |  | other |  | -1.117 | 1.45 |
| TANK |  | other |  |  | 1.45 |
| ZEB2 |  | transcription regulator |  | 0 | 1.45 |
| EBF3 |  | transcription regulator |  |  | 1.45 |
| SS18 |  | transcription regulator |  |  | 1.45 |
| ELF4 |  | transcription regulator |  |  | 1.45 |
| TREM2 |  | transmembrane receptor |  | -1 | 1.45 |
| EBF1 |  | transcription regulator |  | -0.689 | 1.45 |
| SP1 |  | transcription regulator |  | -0.823 | 1.45 |
| WNT5A |  | cytokine |  | -0.218 | 1.44 |
| SPRY2 |  | other |  | 1.342 | 1.44 |
| ISGF3 |  | complex |  |  | 1.43 |
| DHCR24 |  | enzyme |  |  | 1.43 |
| CRY2 |  | enzyme |  |  | 1.43 |
| CRY1 |  | enzyme |  |  | 1.43 |
| CYBB |  | enzyme |  |  | 1.43 |
| Sos |  | group |  |  | 1.43 |
| STAT |  | group |  |  | 1.43 |
| RIPK2 |  | kinase |  | 0.991 | 1.43 |
| PIK3R2 |  | kinase |  |  | 1.43 |
| RPS6KA4 |  | kinase |  |  | 1.43 |
| mir-130 |  | microRNA |  |  | 1.43 |
| ADAP1 |  | other |  |  | 1.43 |
| DANCR |  | other |  |  | 1.43 |
| HLA-A | -2.549 | other |  |  | 1.43 |
| CDC37 |  | other |  |  | 1.43 |
| KPNA2 |  | other |  |  | 1.43 |
| ATXN3 |  | peptidase |  |  | 1.43 |
| PON1 |  | phosphatase |  |  | 1.43 |
| PPP5C |  | phosphatase |  |  | 1.43 |
| DUSP14 |  | phosphatase |  |  | 1.43 |
| SMARCA2 |  | transcription regulator |  |  | 1.43 |
| SMAD7 |  | transcription regulator | Activated | 2.408 | 1.43 |
| SOX5 |  | transcription regulator |  |  | 1.43 |
| PA2G4 |  | transcription regulator |  |  | 1.43 |
| HOXA1 |  | transcription regulator |  |  | 1.43 |
| PRDM16 |  | transcription regulator |  |  | 1.43 |
| RBP1 |  | transporter |  |  | 1.43 |
| MTTP |  | transporter |  |  | 1.43 |
| VEGFA |  | growth factor |  | 0.471 | 1.43 |
| Smad2/3 |  | group |  | -1.067 | 1.43 |
| INSIG1 |  | other | Inhibited | -2 | 1.42 |
